# Behavioural Interventions and Botulinum Toxin Injections for Drooling, Swallowing, Feeding, and Oral-Motor Outcomes in Children: A Domain-Specific Systematic Review and Meta-Analysis of Randomised Controlled Trials

**DOI:** 10.3390/jcm15124653

**Published:** 2026-06-16

**Authors:** Renée Speyer, Jae-Hyun Kim, Lianne Remijn, Karen Malherbe, Belinda Deramore Denver, Deborah Denman, Caleb Anson Davies, Andrea Carrick, Reinie Cordier

**Affiliations:** 1 Discipline of Speech and Language Therapy, School of Health Sciences, College of Medicine, Nursing & Health Sciences, University of Galway, H91 TK33 Galway, Ireland; 2 Curtin School of Allied Health, Faculty of Health Sciences, Curtin University, Perth, WA 6102, Australia; reinie.cordier@curtin.edu.au; 3 Faculty of Medicine, Health and Human Sciences, Macquarie University, Sydney, NSW 2109, Australia; jae-hyun.kim@mq.edu.au; 4 School of Allied Health, HAN University of Applied Sciences, 6503 GL Nijmegen, The Netherlands; lianne.remijn@han.nl; 5 Speech and Language Therapy, Galway University Hospital, H91 YR71 Galway, Ireland; karen.malherbe@hse.ie; 6 School of Allied Health, Faculty of Health Science, Australian Catholic University, Sydney, NSW 2038, Australia; belinda.deramoredenver@acu.edu.au; 7 College of Healthcare Sciences, James Cook University, Townsville, QLD 4811, Australia; deborah.denman@jcu.edu.au; 8 Department of Linguistics, Faculty of Medicine, Health and Human Sciences, Macquarie University, Sydney, NSW 2109, Australia; 9 School of Communities and Education, Faculty of Health and Wellbeing, Northumbria University, Newcastle upon Tyne NE7 7XA, UK; c.a.davies@northumbria.ac.uk (C.A.D.); andrea.carrick@northumbria.ac.uk (A.C.); 10 Manchester Institute of Education, School of Environment, Education and Development, The University of Manchester, Manchester M13 9PL, UK; 11 Department of Health & Rehabilitation Sciences, Faculty of Health Sciences, University of Cape Town, Cape Town 7700, South Africa

**Keywords:** sialorrhoea, dysphagia, paediatric feeding disorder, paediatrics, RCT, rehabilitation, behavioural intervention, botulinum toxin, treatment outcomes

## Abstract

**Objective**: Despite increasing use of behavioural interventions in paediatric swallowing and feeding care, the evidence base remains limited and difficult to interpret due to small sample sizes, heterogeneous interventions, diverse outcome measures, and variability in study populations. This review and meta-analysis, therefore, aimed to evaluate the effects of behavioural interventions and botulinum toxin injections on drooling, swallowing, feeding, and oral-motor outcomes in children, based exclusively on randomised controlled trials (RCTs). **Methods**: Systematic searches were conducted in CINAHL, Embase, and PubMed to identify RCTs. Pharmacological and surgical interventions were excluded, except for botulinum toxin injections, which were analysed as a distinct intervention category given their widespread clinical use in paediatric drooling management. Methodological quality was assessed using the Revised Cochrane Risk of Bias tool (RoB 2). Random-effects meta-analyses were performed, with prediction intervals calculated to account for between-study heterogeneity and to assess the expected range of effects in comparable future studies. **Results**: Twenty-eight studies were included. Behavioural interventions demonstrated moderate-to-large effects on oral-motor outcomes, whereas botulinum toxin injections demonstrated the strongest effects on drooling. Outcomes measured using multi-item caregiver-reported tools showed larger effects. No significant effects were observed for swallowing or feeding outcomes. Effect sizes varied by age, outcome measure, and respondent type, indicating systematic sources of variation in intervention effects. Prediction intervals indicated substantial clinical and methodological variability, suggesting that intervention effects are context-dependent and not consistently generalisable across populations and settings. **Conclusions**: Intervention effectiveness in paediatric swallowing and feeding is domain-specific, with consistent benefits observed for drooling and oral-motor outcomes but not for swallowing or feeding. Outcomes are strongly influenced by the measurement approach. High-quality, standardised RCTs targeting specific functional domains are needed to strengthen the evidence base and inform clinical decision making. However, substantial variability in intervention effects across studies suggests that treatment effectiveness may vary with population characteristics, intervention approaches, and outcome measurement methods.

## 1. Introduction

**Figure 1:**
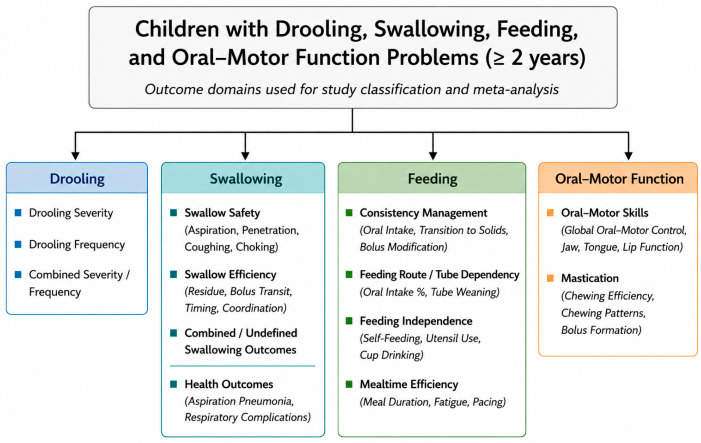
Conceptual framework for classifying drooling, swallowing, feeding, and oral-motor function outcomes in children aged ≥2 years.

Swallowing refers to the transport of a bolus—food, liquid, or saliva—from the oral cavity to the stomach, whereas feeding encompasses a broader set of processes, including breastfeeding or bottle feeding, transitioning to solid foods, and/or the preparation and delivery of food or liquid from a plate or cup to the mouth [1]. Beyond the physical act of swallowing, feeding also involves the wider mealtime context, such as child–caregiver interaction and feeding behaviours [2], which may influence feeding performance and outcomes. To reflect the multidimensional nature of paediatric swallowing and feeding, the authors developed a structured conceptual framework encompassing four functionally related but analytically distinct domains: drooling, swallowing, feeding, and oral-motor function. This framework (Figure 1) supports domain-specific classification of outcomes and evaluation of intervention effects across clinically distinct functional areas.

Difficulties in swallowing and feeding can have serious health consequences, including dehydration, malnutrition, and aspiration pneumonia, and are associated with a high disease burden, substantial psychological and social impacts, and reduced quality of life for both children and caregivers [3,4]. Reflecting the complexity of these disorders, paediatric feeding disorder (PFD) was introduced in 2019 as a diagnostic term describing impaired oral intake that is not age-appropriate and associated with medical, nutritional, feeding-skill, and/or psychosocial dysfunction [3]. Aligned with the International Classification of Functioning, Disability and Health (ICF), this framework recognises the multidimensional nature of paediatric swallowing and feeding disorders and provides a basis for understanding, assessment and management.

Consistent with these multidimensional models, the conceptual framework and its domains capture core components of paediatric swallowing and feeding that may respond differently to intervention. For example, swallowing outcomes may include aspiration or swallowing safety measured using videofluoroscopy, whereas feeding outcomes may involve food acceptance, self-feeding behaviours, or caregiver–child mealtime interaction. Oral-motor outcomes may include chewing efficiency, tongue movement, or mastication skills, while drooling outcomes focus on saliva control and drooling severity. Distinguishing these domains is clinically important, as interventions may target different underlying mechanisms and therefore produce domain-specific effects. The framework provides a consistent basis for categorising outcomes and evaluating domain-specific treatment effects across studies (Figure 1), making it particularly useful for systematic reviews and meta-analyses.

Although several reviews have examined specific aspects of paediatric swallowing and feeding—such as parental stress and feeding practices [5], sensory–behavioural feeding interventions in early childhood [6], oral feeding interventions in pre-term infants [7,8], and swallowing disorders in selected paediatric populations such as children with cerebral palsy [9]—there is currently no domain-specific synthesis that systematically evaluates the highest level of evidence across all four domains of this framework for paediatric swallowing and feeding.

Despite increasing use of behavioural interventions in paediatric swallowing and feeding care, the evidence base remains limited and challenging to interpret due to small sample sizes, diverse intervention approaches, diverse outcome measures, and variability in study populations. In contrast, a more substantial body of evidence exists for adults with dysphagia, where systematic reviews have focused on randomised controlled trials (RCTs) to synthesise the highest level of evidence [4,10,11]. Consistent with this approach, and acknowledging the smaller volume of paediatric RCTs, the present review focuses exclusively on RCTs to identify the strongest available evidence and highlight gaps in the literature. In addition to synthesising intervention effects, the review was intended to help identify methodological and conceptual considerations relevant to the design of future paediatric swallowing and feeding trials.

The conceptual framework informed the analytical strategy by guiding outcome classification, synthesis across domains, meta-analytic grouping, and subgroup analyses. Outcomes were assigned to domains according to the primary construct measured by the assessment method used in each study. Because botulinum toxin injections represent a distinct and widely used intervention for drooling, they were included as a separate intervention category to enable comparison across intervention types within this domain. However, to avoid the scope of the review becoming too broad, all other medical and surgical interventions were excluded. This systematic review and meta-analysis aimed to evaluate the effects of behavioural interventions on swallowing, feeding, and oral-motor outcomes, as well as the effects of botulinum toxin (Botox) injections on drooling in children, using evidence derived solely from RCTs. Intervention effects were examined across the four outcome domains to determine whether effectiveness varied by functional area. Random-effects meta-analysis with prediction intervals was used to quantify intervention effects and between-study heterogeneity. Subgroup analyses were conducted to explore factors that may influence outcomes, including age group, diagnostic group, respondent type, outcome measurement method, intervention type, and study quality.

## 2. Methods

This systematic review followed the Preferred Reporting Items for Systematic Reviews and Meta-Analyses (PRISMA) guidelines. The PRISMA 2020 statement and checklist (Supplementary Tables S1 and S2) aim to ensure comprehensive and transparent reporting of systematic reviews [12,13]. The review protocol was developed a priori and registered with PROSPERO (CRD42024581171).

### 2.1. Information Sources

Studies were identified through literature searches conducted on 11 May 2025 across three electronic databases: CINAHL Ultimate, Embase, and PubMed. Supplementary searches involved reviewing the reference lists of eligible articles.

### 2.2. Search Strategies

Electronic searches were conducted in all three databases using both controlled vocabulary terms (i.e., MeSH in PubMed, Emtree in Embase, and CINAHL Headings in CINAHL Ultimate) and free-text terms. Two sets of terms were combined: (1) those related to drooling, swallowing, and feeding, and (2) terms associated with randomised controlled trials. The complete electronic search strategies are presented in Table 1.

### 2.3. Eligibility Criteria

To qualify for inclusion in this review, studies were required to meet the following criteria: (1) studies included children with drooling, swallowing, and/or feeding problems as described by Speyer et al. (2019) [14]. Drooling refers to the involuntary loss of saliva, liquid, or food from the oral cavity due to incomplete lip closure. Feeding and swallowing problems were considered broadly and included, but were not limited to, dysphagia, feeding difficulties, impairments in self-feeding, oral-motor dysfunction and masticatory problems. Swallowing problems specifically involve impairments in the transport of a bolus from the oral cavity to the stomach and may be characterised by poor tongue function, delayed initiation of swallowing, or reduced pharyngeal motility.

Feeding problems encompass difficulties with breastfeeding or bottle feeding, transitioning to solid foods, and/or the processes involved in preparing, arranging, and bringing food or liquid from a plate or cup to the mouth. These problems included both child–caregiver interactions and child behaviours and could manifest as prolonged feeding times or delayed development of oral feeding skills. (2) Studies examined drooling, swallowing, and/or feeding interventions and could include a wide range of treatments, such as behavioural approaches (e.g., oral-motor training), acupuncture, or neurostimulation. Pharmacological and surgical interventions were excluded, except for botulinum toxin injections, which were included in a distinct intervention category given their widespread clinical use as a first-line treatment for paediatric drooling. Botulinum toxin injections were not classified as behavioural interventions but were analysed separately within drooling outcomes to enable comparison across intervention types while maintaining conceptual clarity regarding mechanisms of action. (3) Studies were randomised controlled trials (RCTs). (4) Studies included a comparison group. (4) Participants were randomly assigned to one of the study arms or groups. (5) The mean age of the children was 18 years or younger, with a minimum age of 2 years at baseline or randomisation.

The lower age limit was chosen because, by two years of age, most children have established fundamental feeding skills—including self-feeding, chewing, and coordinated swallowing—allowing persistent feeding difficulties to be distinguished from transient developmental variations [15]. Restricting the review to children aged 2 years or older may reduce the influence of developmental factors more specific to neonates and infants (e.g., rapid growth and early oral-motor maturation), ensure greater consistency in intervention targets and outcomes, and allow the review to focus specifically on children as a distinct developmental population with age-specific intervention targets and outcomes. (6) Only original peer-reviewed articles were included, excluding conference abstracts, reviews, case reports, student dissertations, and editorials. (7) No restrictions were applied regarding the language of the publications.

Studies exclusively reporting on malocclusion, dental caries, dysarthria, dyspraxia, or weight loss were excluded. Likewise, studies focusing on oesophageal problems (e.g., regurgitation, vomiting, reflux), eating disorders (e.g., anorexia, bulimia), or behavioural eating aversions and picky eating were considered beyond the scope of this review. Outcomes not directly related to the child, such as parental stress or quality of life, were also excluded.

### 2.4. Systematic Review

***Methodological Quality and Risk of Bias***. The methodological quality of included studies was assessed using the Revised Cochrane Risk of Bias tool for randomised trials (RoB 2) at the outcome level [16], which evaluates potential bias across five domains: (1) the randomisation process, (2) deviations from intended interventions, (3) missing outcome data, (4) outcome measurement, and (5) selection of reported results.

***Data Collection Process***. A standardised data extraction form was developed to collect information from the included studies across the following categories: study country, participant diagnosis, inclusion and exclusion criteria, sample size, age, gender, intervention goal, intervention agent, dosage and timing of measurements, intervention materials and procedures, outcome measures, and treatment outcomes. Two authors independently extracted the data, with any disagreements resolved by consensus with a third author.

***Data, Items and Synthesis of Results***. All retrieved records published in English were imported into Research Screener, a machine-learning-assisted citation screening tool that ranks records by predicted relevance [17]. Prior to screening, a seed file of known relevant studies selected by the authors was uploaded to train the system. Research Screener subsequently used machine learning based on this seed file to identify and rank similar studies retrieved from the search strategies according to their predicted relevance.

Titles and abstracts were independently screened by two authors, with priority given to the highest-ranked records. This approach enabled efficient identification of potentially relevant studies while ensuring comprehensive coverage. Screening continued until no additional records were considered relevant. Records published in languages other than English were screened separately, as Research Screener only supports English-language records.

Both reviewers subsequently assessed the selected full-text articles for eligibility, with any discrepancies resolved through discussion or, when necessary, consultation with a third author to reach consensus. Where the authors were not proficient in the language in which the studies were published, online DeepL translation services were consulted.

Methodological quality was independently assessed by two researchers, with a third reviewer consulted as needed to resolve disagreements. Reviewers had no affiliations with the authors of the included studies, minimising potential bias. Study data were extracted using comprehensive data collection forms, risk of bias was evaluated using RoB 2 [16], and treatment outcomes were summarised using effect sizes and statistical significance.

### 2.5. Meta-Analysis

Data were extracted from eligible studies to compare between-group effect sizes for post-intervention outcomes related to drooling, swallowing, feeding, and oral-motor function. The unit of analysis was the study-level comparison between experimental and comparison groups. Outcome measures were categorised into four domains in accordance with the conceptual framework. The first domain, drooling, included measures of drooling severity and/or frequency. The second domain, swallowing, comprised outcomes related to swallowing safety (e.g., aspiration, penetration, coughing, or choking), swallowing efficiency (e.g., residue, bolus transit times, timing, and coordination), combined safety–efficiency measures or undefined swallowing measures, and swallowing-related health outcomes (e.g., incidence of aspiration pneumonia, respiratory complications, or hospitalisations). The third domain, feeding, included consistency management (e.g., oral intake, transition to solids, bolus modification), feeding route and tube dependency, feeding independence (e.g., level of assistance, self-feeding skills, utensil use), and mealtime efficiency (e.g., mealtime duration). The fourth domain, oral-motor function, encompassed global oral-motor skills, jaw, tongue, and lip function, as well as masticatory outcomes, including chewing efficiency, chewing patterns, and bolus formation.

When multiple outcome measures within a study assessed the same domain, a single outcome was selected to avoid statistical dependency, prioritising the authors’ primary outcome or the most widely used validated measure. In some cases, the primary outcome addressed a broader or different study aim, and the reported measure that most directly reflected drooling, feeding, swallowing, or oral-motor outcomes relevant to this review was therefore selected. This approach ensured that each study contributed only one independent effect size per outcome domain to the meta-analysis, consistent with meta-analytic methodology, which requires statistical independence of observations. Including multiple outcomes from the same study in a single analysis would give that study disproportionate weight, as the same participants would effectively be counted multiple times, potentially biasing pooled effect estimates and confidence intervals. Therefore, when several outcomes in the same domain were reported in a single study, one representative outcome was selected according to predefined criteria to ensure methodological consistency, facilitate greater comparability, and reduce heterogeneity across studies.

To compare effect sizes, group means, standard deviations, and sample sizes for the experimental and comparison groups at post-intervention, data were entered into Comprehensive Meta-Analysis (CMA) software, version 4 [18]. When only non-parametric data were reported (i.e., medians and interquartile ranges), these were converted to parametric estimates using established statistical transformation methods for inclusion in the meta-analysis. For trials with multiple intervention arms, comparisons were structured to avoid double-counting participants by combining relevant intervention groups or selecting the most appropriate comparison group. For studies with insufficient data for meta-analysis, corresponding authors were contacted via email to request additional information when required data were missing or incomplete.

Effect sizes were calculated in Comprehensive Meta-Analysis (CMA) using a random-effects model to account for expected heterogeneity across studies in participant characteristics, interventions, and outcome measures. Under this model, the true intervention effects were assumed to vary across studies rather than representing a single common effect size, reflecting expected clinical and methodological heterogeneity. Random-effects models were selected a priori due to expected clinical and methodological heterogeneity across paediatric populations, interventions, and outcome measures. Between-study heterogeneity was quantified using the between-study variance (τ^2^). To provide a clinically interpretable estimate of heterogeneity, 95% prediction intervals were calculated to estimate the range within which the true effect of a similar future study is expected to fall [19]. Standardised mean differences were calculated using Hedges’ *g* with 95% confidence intervals. Positive effect sizes indicated improvement favouring the experimental intervention. Effect sizes were interpreted using Cohen’s conventions: *g* ≈ 0.20 (small effect), *g* ≈ 0.50 (moderate effect), and *g* ≥ 0.80 (large effect) [20].

Forest plots of effect sizes were generated for each outcome domain and subdomain. Behavioural interventions (for drooling, swallowing, feeding, and oral-motor outcomes) and botulinum toxin injections (for drooling outcomes only) were compared with alternative treatments, no-treatment controls, or placebo/sham interventions, with intervention types analysed according to their relevant outcome domains. Subgroup analyses were conducted to examine differences in effect sizes according to medical diagnoses, age group (2–5, 6–11, and 12–18 years), respondent type (clinician, caregiver, or teacher), outcome measure (single-item or multiple-item), intervention type, and methodological study quality as assessed using RoB 2. These subgroup analyses were considered exploratory due to the limited number of studies in some categories.

Publication bias was assessed using Comprehensive Meta-Analysis (CMA) software through Begg and Mazumdar’s rank correlation test and Rosenthal’s fail-safe N. The Begg and Mazumdar test evaluates the rank correlation between standardised effect sizes and their variances [21], producing a tau statistic and a two-tailed *p*-value. A tau value of zero indicates no relationship, whereas deviations from zero suggest a potential association; in cases of publication bias, studies with larger standard errors tend to report larger effect sizes. A positive tau indicates larger effects are associated with smaller variances, while a negative tau indicates larger effects are associated with larger variances. The fail-safe N estimates the number of additional studies with an effect size of zero that would be required to render the meta-analytic result non-significant [22]. A small fail-safe N raises concern about the robustness of the effect, whereas a large fail-safe N indicates that, although the effect may be somewhat inflated due to missing studies, it is unlikely to be null.

Prediction intervals were also calculated in CMA to estimate the range within which the effect of a similar future study is likely to fall, complementing the primary meta-analytic estimates. Unlike confidence intervals, which reflect uncertainty around the average effect, prediction intervals incorporate between-study heterogeneity (*τ*^2^) and provide a more realistic estimate of variability across contexts. Prediction intervals were prioritised because they provide a clinically interpretable estimate of the dispersion of true effects across heterogeneous studies. These intervals complement, but do not replace, traditional publication-bias assessments such as the fail-safe N.

## 3. Results

### 3.1. Study Selection

A total of 10,574 records were identified through database searching in CINAHL (*n* = 707), Embase (*n* = 1849), and PubMed (*n* = 8018). After removal of duplicates (*n* = 654), 9920 records remained for screening. Records without authors (*n* = 25) or without an abstract (*n* = 321) were excluded prior to screening. Records published in languages other than English (*n* = 220) were assessed manually. The remaining records (*n* = 9354) were imported into Research Screener for title and abstract screening.

**Figure 2:**
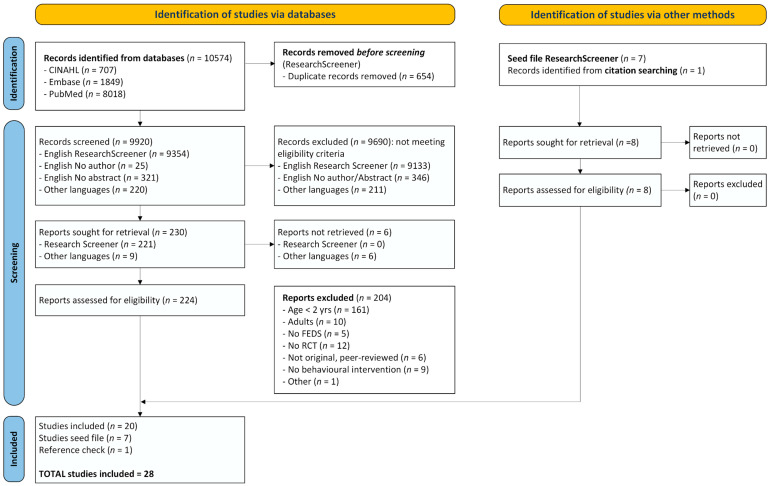
Flow diagram of the reviewing process according to PRISMA [12,13].

Following title and abstract screening, 230 full-text articles were retrieved. Of these, 20 studies met the inclusion criteria. An additional six studies were identified from the Research Screener seed file, and one study was identified through reference checking of included studies. In total, 28 studies were included in the systematic review. Figure 2 presents the PRISMA 2020 flow diagram of the study selection process. Of the 28 included studies, a subset contributed to each outcome domain meta-analysis (drooling, swallowing, feeding, and oral-motor), with the number of studies (k) varying by domain (drooling *k* = 3, swallowing *k* = 13, feeding *k* = 9, oral-motor *k* = 6). Results are presented according to the four predefined functional domains of the conceptual framework (drooling, swallowing, feeding, and oral-motor function), with study characteristics and intervention details summarised separately to support interpretability across heterogeneous interventions and populations.

### 3.2. Description of Studies

All 28 included studies are described in detail in Supplementary Tables S3 and S4 [23,24,25,26,27,28,29,30,31,32,33,34,35,36,37,38,39,40,41,42,43,44,45,46,47,48,49,50,51,52,53]. Table S3 summarises study characteristics and the definitions and methods used to identify and diagnose drooling, swallowing, and feeding problems. It also provides detailed information on participant groups, including medical diagnoses, sample sizes, ages, and genders across all study groups. Table S4 presents intervention goals, intervention components (such as intervention agent, dosage, and measurement time points), intervention materials and procedures, outcome measures, and treatment outcomes for each included study.

#### 3.2.1. Participants

The 28 studies included a total of 1312 participants (mean = 49; SD = 42.062), with the sample sizes across studies ranging from 6 [40] to 218 participants [31]. The mean age of participants in the experimental groups was 6.7 years (SD = 3.067), and the mean proportion of boys across all studies was 54.5% (SD = 13.058). Most studies included children with cerebral palsy (*n* = 19), while several studies (*n* = 7) included children with a variety of medical diagnoses, such as non-progressive neurodevelopmental disabilities or intellectual disabilities [29,30,31,39,41,42,47], Down syndrome (*n* = 1 [50]) or orofacial dysfunction without a specified underlying medical diagnosis (*n* = 1 [37]). The included studies were conducted across fourteen countries, with the largest number conducted in Turkey (*n* = 8), followed by Canada (*n* = 3), Germany (*n* = 3), Iran (*n* = 3), and the Netherlands (*n* = 2). The remaining studies (*n* = 8) were conducted in China, Egypt, Italy, Jordan, Norway, Pakistan, South Korea, and Taiwan.

#### 3.2.2. Outcome Measures

A wide variety of outcome measures were used across the included studies. Drooling problems were most frequently assessed using the Thomas–Stonell Drooling Rating Scale or closely related measures [26,27,28,32,36,38,49] or a drooling quotient [29,38,40,49]. Other studies employed the Drooling Impact Scale [27,42], the Teacher Drooling Scale [41,47], salivary flow rates [31,40], or study-specific caregiver measures [30,47].

Only two studies included instrumental assessments when evaluating swallowing problems: flexible fiberoptic endoscopic evaluation of swallowing (FEES) [46] or videofluoroscopic swallowing study (VFSS) [33]. Other measures reported across studies included the Pediatric Eating Assessment Tool (Pedi-Eat) [39], the Screen Tool for Eating/Feeding Problems (STEP) [50], the Behavioral Assessment of Severe Oral Feeding Difficulties (BASOFF; item “Swallowing without cough”) [45], and the modified Functional Feeding Assessment (FFA; domain “Clearing”) [35].

Feeding problems were reported using the ASHA National Outcome Measurement System (ASHA NOMS) [45], the Functional Oral Intake Scale (FOIS) [25], selected items from the Schedule for Oral Motor Assessment (SOMA; consistency management—items “Puree”, “Semi-solid”, “Solids” and “Cracker”—and item “Cup”) [24,39], a selected item from the modified Functional Feeding Assessment (FFA; domain “cup drinking”) [24], and mealtime duration [25,33,34].

When targeting oral-motor skills more broadly, studies used the total scores of the Behavioural Assessment Scale of Oral Functions in Feeding (BASOFF) [45], the Oral Motor Assessment Scale (OMAS) [39], or the Schedule for Oral Motor Assessment (SOMA) [39]. Mastication was most frequently assessed using the Karaduman Chewing Performance Scale (KCPS) [32,36,43] or items from measures such as the Behavioural Assessment Scale of Oral Functions in Feeding (BASOFF: item “Chewing food”) [45], the modified Functional Feeding Assessment (FFA: domain “Chewing”) [35], and the Oral Motor Assessment Scale (OMAS: item “Mastication”) [23]. Tongue movement was assessed using the Tongue Thrust Rating Scale (TTRS) [32,36] or a single Behavioural Assessment Scale of Oral Functions in Feeding item (BASOFF; item “Tongue control”) [45].

Further details on the outcome measures are provided in Supplementary Table S4. The table summarises the measures and specific items used across the included studies, where applicable.

#### 3.2.3. Interventions

The 28 included studies reported behavioural interventions and botulinum toxin injections delivered by physiotherapists (*n =* 6), speech–language therapists (*n* = 3), occupational therapists (*n* = 2), feeding assistants (*n* = 2), and physicians from diverse backgrounds (*n* = 5). One study trained parents to deliver the intervention, and four studies involved collaboration with parents or caregivers. Nine studies did not specify the intervention agents, and four studies involved more than one professional discipline. Intervention dosage varied widely, ranging from single-session treatments (e.g., botulinum toxin injections) to exercise programs lasting up to six months [44].

Of the 28 RCTs, the majority included two groups, while three studies [33,34,49] included three groups, and one study included four groups [28]. Most studies compared an experimental intervention with usual care (e.g., traditional or standard care) or with similar interventions. A smaller number of studies compared an experimental group with either a placebo intervention (i.e., saline injection [26,31,48]) or sham intervention [25,38,39,45,46,49], while others used a no-treatment control group [28,33,34,42,49].

#### 3.2.4. Framework

Based on the outcome measures used, studies were categorised into a conceptual framework comprising four domains: drooling, swallowing, feeding, and oral-motor functions (Table 2). The drooling domain included measures of drooling severity, frequency, combined severity and frequency, and other related assessments. Swallowing outcomes were grouped under swallow safety (e.g., penetration and/or aspiration, coughing, and choking), swallow efficiency (e.g., bolus residue, bolus transit times, swallow timing and coordination), combined measures of safety and efficiency, and health outcomes (e.g., incidence of aspiration pneumonia, respiratory complications, and hospitalisations). Feeding outcomes encompassed consistency management (e.g., oral intake, transition to solids, bolus modification), feeding route and tube dependency (e.g., percentage of oral intake, duration of tube use, weaning success), feeding independence (e.g., level of assistance, self-feeding skills, utensil use, cup drinking), and mealtime efficiency (e.g., mealtime duration). The oral-motor functions domain included global oral-motor skills (e.g., bite–chew–swallow coordination, OMAS total score) as well as jaw, tongue, and lip function (e.g., strength, range of motion, and coordination). As several studies specifically targeted chewing, a separate mastication category was also created (e.g., chewing efficiency and patterns). Groupings of studies were determined by consensus among the authors, recognising that individual studies could be assigned to more than one domain or category depending on the outcome measures used. This framework also provided a structured basis for the meta-analyses, enabling outcomes to be grouped consistently and compared across studies.

### 3.3. Methodological Quality

**Figure 3:**
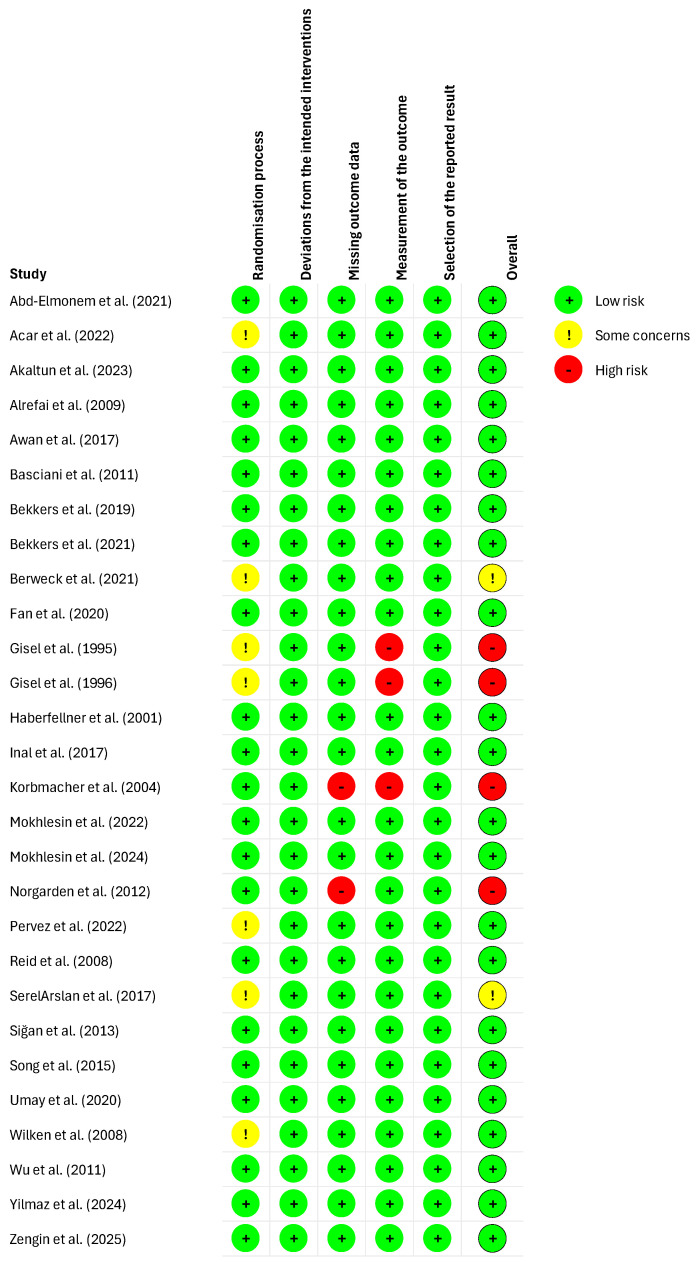
Risk-of-bias summary for individual studies (*N* = 28) [23,24,25,26,27,28,29,30,31,32,33,34,35,36,37,38,39,40,41,42,43,44,45,46,47,48,49,50] in accordance with RoB 2 [16].

**Figure 4:**
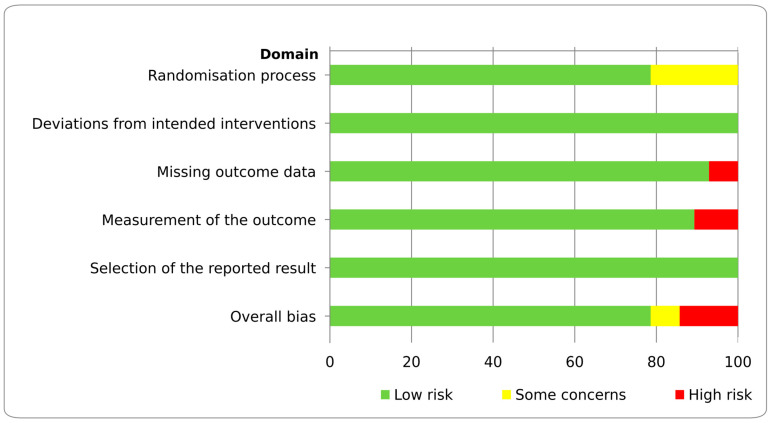
Risk-of-Bias Summary (RoB 2, *N* = 28).

The risk of bias in the included RCTs was assessed using the RoB 2 tool. Figure 3 and Figure 4 present the risk-of-bias summary per domain for individual studies and across all included studies. Most studies (22/28) demonstrated low risk of bias per domain; two studies were rated as having some concerns [31,43], and four studies were identified as being at high risk [33,34,37,40].

### 3.4. Risk-of-Bias Assessment

The Begg and Mazumdar rank correlation test produced a tau of 0.410 (two-tailed *p* = 0.820), indicating no evidence of publication bias. Publication bias analyses were conducted across the pooled meta-analytic dataset. This meta-analysis included data from 18 studies, yielding a combined *z*-value of 7.495 (two-tailed *p* < 0.001). The fail-safe N was 246, meaning that 246 null studies would need to be identified and included for the overall two-tailed *p*-value to exceed 0.050. In other words, approximately 13.7 missing studies would be required for every observed study to nullify the effect. Both the Begg and Mazumdar rank correlation and the fail-safe N indicate no evidence of publication bias.

### 3.5. Meta-Analysis: Effect of Interventions

**Figure 5:**
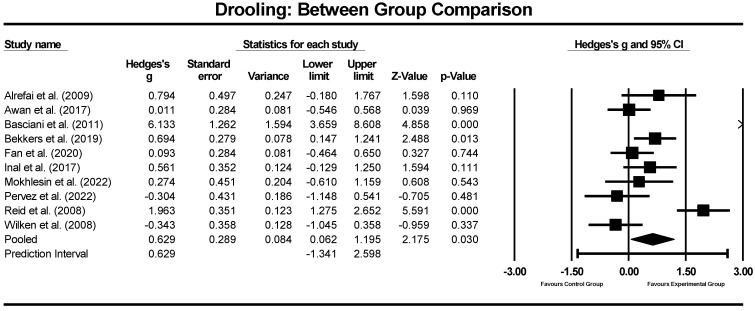
Meta-analysis forest plot of post-intervention drooling [26,27,28,29,32,36,38,41,42,47].

**Figure 6:**
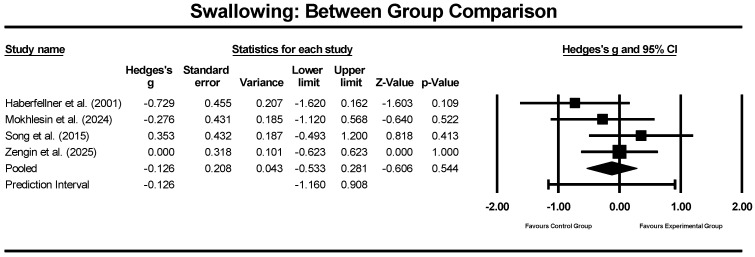
Meta-analysis forest plot of post-intervention swallowing [35,39,45,50].

**Figure 7:**
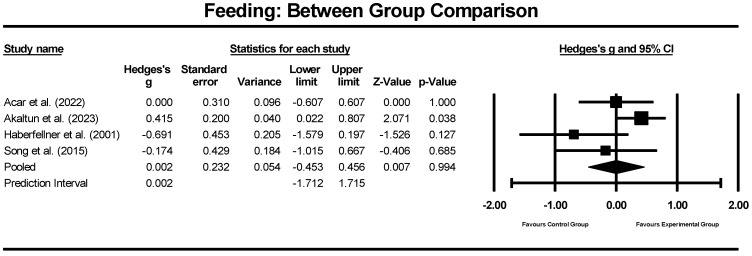
Meta-analysis forest plot of post-intervention feeding [24,25,35,45].

**Figure 8:**
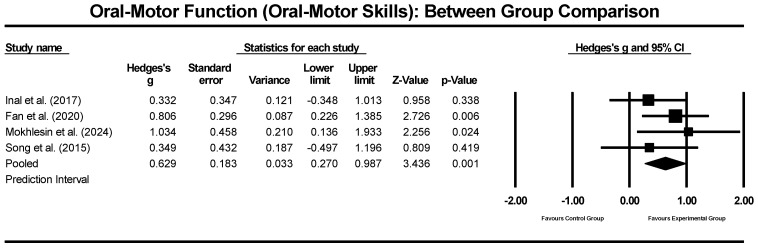
Meta-analysis forest plot of post-intervention oral-motor skills [32,36,39,45].

**Figure 9:**
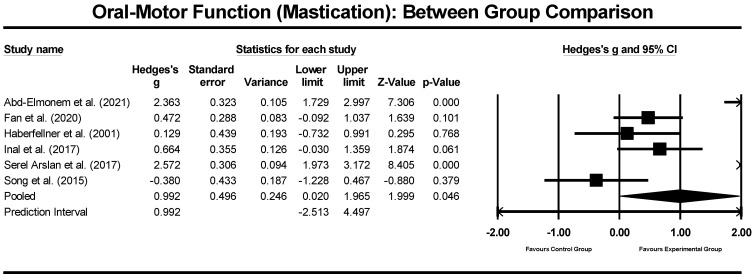
Meta-analysis forest plot of post-intervention mastication [23,32,35,36,43,45].

Of the 28 randomised controlled clinical trials, 18 studies were included in the meta-analysis [23,24,25,26,27,28,29,32,35,36,38,39,41,42,43,45,47,50]. Ten studies were excluded for various reasons: four were rated as high risk of bias using the RoB 2 tool [33,34,37,40], five provided insufficient data for meta-analysis [31,44,46,48,49], and two reported on the same participant group [29,30], resulting in the exclusion of the latter study [30]. Meta-analyses were conducted across four outcome domains: drooling, swallowing, feeding, and oral-motor functions. Oral-motor functions were further subdivided into oral-motor skills and mastication. Summary effect estimates for each domain are presented in Table 3 and illustrated in Figure 5, Figure 6, Figure 7, Figure 8 and Figure 9.

#### 3.5.1. Between Group Analyses

Meta-analyses compared experimental and control groups across four domains (Table 3, Figure 5, Figure 6, Figure 7, Figure 8 and Figure 9): drooling, swallowing, feeding, and oral-motor functions (the latter subdivided into oral-motor skills and mastication). Using a random-effects model, a significant approximately moderate post-intervention effect was found for drooling (*z* = 2.175, *p* = 0.030, Hedges’ *g* = 0.629, and 95% CI = 0.062–1.195, prediction interval −1.341 to 2.598, τ^2^ = 0.646), while swallowing (*z* = −0.606, *p* = 0.544, Hedges’ *g* = −0.126, and 95% CI = −0.533–0.281, prediction interval −1.160 to 0.908, τ^2^ = 0.014) and feeding (*z* = 0.007, *p* = 0.994, Hedges’ *g* = 0.002, and 95% CI = −0.453–0.456, prediction interval −1.712 to 1.715, τ^2^ = 0.105) showed no significant effects. Within oral-motor function, both oral-motor skills and mastication demonstrated significant effects, with approximately moderate (*z* = 3.436, *p* < 0.001, Hedges’ *g* = 0.629, and 95% CI = 0.270–0.987; τ^2^ = 0) and large intervention effect sizes (*z* = 1.999, *p* = 0.046, Hedges’ *g* = 0.992, and 95% CI = 0.020–1.965, prediction interval −2.513 to 4.497, τ^2^ = 1.348), respectively.

Intervention effects varied widely across studies, ranging from 0.000 to 6.133 overall: drooling, 0.011 to 6.133; swallowing, 0.000 to 0.729; feeding, 0.000 to 0.691; oral-motor skills, 0.349 to 1.034; and mastication, 0.129 to 2.572. Among the ten intervention groups targeting drooling, four targeting swallowing, four targeting feeding, four targeting oral-motor skills, and six intervention groups across three studies targeting mastication, seven groups demonstrated large effect sizes (Hedges’ *g* > 0.8), six groups demonstrated moderate effect sizes (0.5 < Hedges’ *g* ≤ 0.8), nine groups demonstrated small effect sizes (0.2 < Hedges’ *g* ≤ 0.5), and six groups demonstrated negligible effect sizes (Hedges’ *g* ≤ 0.2).

Between-study heterogeneity was significant for both drooling (*Q*(9) = 52.267, *p* < 0.001) and mastication (*Q*(5) = 60.966, *p* < 0.001), with wide prediction intervals indicating substantial variability in the range of intervention effects that may be observed in similar future studies. In contrast, no significant heterogeneity was observed for swallowing (*Q*(3) = 3.268, *p* = 0.352), feeding (*Q*(3) = 5.986, *p* = 0.112), and oral-motor skills (*Q*(3) = 2.288, *p* = 0.515), with prediction intervals indicating comparatively limited variability in effect estimates across studies. These prediction intervals reflect the expected range of intervention effects that might be observed in future studies, accounting for between-study heterogeneity across paediatric populations, interventions, and outcome measures. Prediction intervals are particularly informative in this context because they estimate the range of effects that might be expected in future studies, accounting for the substantial clinical and methodological heterogeneity across paediatric populations, interventions, and outcome measures.

#### 3.5.2. Between-Subgroup Analyses

Where data were available, subgroup analyses using a mixed-effects model examined differences in effect sizes by outcome measure (single- vs. multiple-item), respondent type (caregiver, clinician, or teacher), diagnostic group (cerebral palsy, Down syndrome, or mixed population), age group (2–5 vs. 6–11 years), and RoB 2 scores (see Table 4), with effects interpreted within outcome domains. In these analyses, effect sizes were pooled within subgroups using a random-effects model, while differences between subgroups were tested using fixed-effects comparisons. Subgroup analyses should be interpreted cautiously because several categories included only one study.

For studies targeting drooling, significantly large intervention effects were observed, with the strongest effects associated with botulinum toxin injections, favouring multiple-item over single-item outcome measures. Significant effects were also found across respondent types, with larger effects for caregiver-reported outcomes and moderate effects for clinician-reported outcomes. Regarding age groups, interventions in older children (6–11 years) showed large effects, whereas no significant effects were observed in the younger group (2–5 years). No significant effects were identified across diagnostic groups. When comparing intervention types within drooling outcomes, botulinum toxin injections demonstrated significantly larger effects. In contrast, studies comparing two botulinum toxin treatment conditions (e.g., injection site or toxin type) did not identify significant differences, nor were significant effects observed for other intervention comparisons.

In contrast, no significant effects were observed in subgroup analyses for studies reporting on swallowing or feeding outcomes. For studies on oral-motor skills, behavioural intervention effects were significant for both multiple-item and single-item outcome measures, with large effects for multiple-item measures and moderate effects for single-item measures. Age effects were observed in both oral-motor skills and mastication studies: significant moderate effects were found in both age groups for oral-motor skills, whereas in mastication studies, significant moderate effects were observed only in the younger group.

Subgroup analyses based on methodological quality (RoB 2) were also conducted for studies examining mastication outcomes. Studies rated as having a low risk of bias demonstrated a non-significant effect. In contrast, the single study rated as having some concerns showed a large and statistically significant effect. Given the small number of studies within these subgroups, these findings should be interpreted with caution.

## 4. Discussion

This systematic review aimed to summarise the effects of behavioural interventions and botulinum toxin injections in managing drooling, swallowing, and feeding problems and to evaluate whether the effectiveness of interventions differs across functional outcome domains. Only the highest level of evidence—namely, RCTs—was included. Findings were reported in accordance with PRISMA guidelines and synthesised using meta-analytic procedures.

### 4.1. Systematic Review Findings

In total, 28 RCTs investigated the effects of behavioural interventions and botulinum toxin injections in children with drooling, swallowing, and feeding problems, providing the highest level of evidence available for evaluating the effectiveness of interventions in this field. Overall, the number of high-quality trials in paediatric populations remains limited. By comparison, systematic reviews of randomised controlled trials in adults with dysphagia (e.g., [4,10,11]) included substantially larger bodies of evidence. This contrast underscores a significant gap in the evidence base for children and highlights the need for further high-quality research in paediatric populations. In addition, the majority of included studies focused on children with cerebral palsy, which may limit the generalisability of the findings to broader paediatric populations with swallowing and feeding disorders. The record selection process for this review revealed that the vast majority of studies focused on very young children, typically under two years of age, and often acute care settings. In contrast, relatively few studies examined long-term follow-up outcomes in these early populations, and even fewer targeted older children more broadly. This imbalance suggests a strong emphasis on early-stage or acute management, with limited attention to the longer-term effects of interventions and developmental trajectories across childhood. Furthermore, none of the included studies reported on clinically meaningful health outcomes such as the incidence of aspiration or pneumonia, respiratory complications, or related hospitalisations, thereby limiting insight into the broader medical impact of these interventions.

To facilitate the evaluation and reporting of intervention effects, a conceptual framework was developed encompassing the domains of drooling, swallowing, feeding, and oral-motor function, enabling domain-specific analysis of intervention effectiveness. Within this framework, oral-motor function was further divided into subdomains, such as oral-motor skills and mastication, enabling a more comprehensive and systematic classification of outcomes. In 2019, the diagnostic term paediatric feeding disorder (PFD) was introduced [3] and defined as impaired oral intake that is not age-appropriate and is associated with medical, nutritional, feeding-skill, and/or psychosocial dysfunction. This definition aligns with the International Classification of Functioning, Disability and Health (ICF) and includes specific diagnostic criteria for PFD. However, most studies in the existing literature did not report these diagnostic criteria, nor did they classify study populations according to the ICF. Therefore, outcomes in this review are presented according to the domains and subdomains defined in the conceptual framework, rather than being restricted to formal diagnostic categories. This approach also allows for separate meta-analyses for each subdomain, enabling a more focused and meaningful synthesis of intervention effects, and informed the decision to exclude outcomes not directly related to the child—such as parental stress or quality of life—which are considered part of psychosocial dysfunction in PFD.

The included studies employed a wide variety of outcome measures to assess different aspects of treatment effects, with findings indicating that intervention effects are strongly influenced by the type and structure of outcome measurement, representing a key source of variability across studies and a critical limitation in the current evidence base. These findings suggest that differences in reported effectiveness may, in part, reflect measurement artefacts rather than true differences in intervention impact, highlighting the need for standardised, domain-specific outcome measures. The absence of standardised outcome measures in paediatric swallowing and feeding research complicates direct comparisons between trials and reduces the precision of pooled estimates in meta-analysis. Several studies relied on single-item indicators or study-specific measures rather than validated multidimensional tools, which may limit sensitivity to change and contribute to variability in reported intervention effects. The development and consistent use of psychometrically robust, domain-specific outcome measures is therefore essential for advancing evidence-based practice in paediatric dysphagia research [14].

### 4.2. Meta-Analysis Findings

Behavioural interventions appear effective in improving oral-motor function in children, while botulinum toxin injections demonstrate the strongest effects in reducing drooling, demonstrating that intervention effectiveness is domain-specific, with moderate-to-large effects observed for oral-motor skills, mastication, and salivary control. In contrast, swallowing and feeding showed no significant improvements, indicating that current evidence does not demonstrate consistent effectiveness of intervention approaches in these core areas of clinical practice and likely reflecting both the limited number of available trials and the complex physiological mechanisms underlying these functions, including the integration of sensory, motor, and respiratory processes, which may be less responsive to behavioural interventions that primarily target isolated components, thereby limiting the likelihood of measurable change. Swallowing is a highly coordinated sensorimotor behaviour involving the oral, pharyngeal, and respiratory systems, and improvements may require multimodal interventions that target physiological impairments rather than behavioural approaches alone. Similarly, feeding difficulties in children often arise from a combination of medical, nutritional, feeding-skill, and psychosocial factors, as reflected in the multidimensional framework of paediatric feeding disorder (PFD) [3]. Interventions that address only one aspect of this complex system may therefore produce limited measurable change in feeding outcomes, particularly when outcome measures focus on isolated behavioural indicators rather than broader functional improvements.

Intervention effects varied across studies and outcome domains and were influenced by factors such as age, outcome measure type, and respondent. Botulinum toxin injections, particularly in studies involving older children and using caregiver-reported, multi-item measures, demonstrated the strongest effects on drooling outcomes. In contrast, improvements in mastication were most evident in younger children, which may reflect developmental differences in the age at which feeding-related skills are acquired. In particular, this pattern may be associated with greater neuroplasticity and the developmental responsiveness of chewing skills during early childhood, supporting the importance of early intervention to optimise functional outcomes. Chewing skills develop rapidly during early childhood as children transition to more complex food textures, and targeted training during this developmental window may therefore produce larger functional gains. Early intervention may also capitalise on heightened neuroplasticity within sensorimotor networks responsible for oral-motor control, facilitating improvements in chewing coordination and efficiency. Previous paediatric dysphagia research similarly suggests that skill-based oral-motor training may be particularly relevant during periods of developmental change [33,54]. Although botulinum toxin injections are pharmacological in nature, they were included in the present review as a distinct intervention category because they are frequently delivered within multidisciplinary management programs for drooling in children with neurodevelopmental disorders. In clinical practice, botulinum toxin is often combined with behavioural or rehabilitative strategies to improve oral-motor control and salivary management. Including these interventions, therefore, reflects real-world clinical practice and allows comparison of behavioural interventions and botulinum toxin as distinct but complementary approaches within the same evidence framework.

Substantial heterogeneity was observed across interventions, paediatric populations (by age and medical diagnoses), and outcome measures, with prediction intervals indicating that intervention effects are context-dependent and not consistently generalisable across settings. The variability likely reflects differences in intervention intensity, treatment fidelity, and underlying neurodevelopmental profiles across study populations. In addition, much of the current evidence base is dominated by studies involving children with cerebral palsy, which further limits the generalisability of the findings to other paediatric populations with swallowing and feeding disorders. Although subgroup analyses did not identify statistically significant differences in treatment effects between cerebral palsy and non-cerebral palsy populations, this finding should be interpreted with caution due to the limited number of studies involving non-cerebral palsy populations.

This variability underscores the need for standardised, psychometrically robust measures and more rigorous study designs, including larger sample sizes that allow for meaningful subgroup comparisons and more reliable meta-analyses. Accordingly, the findings of the present review—particularly the pooled estimates—should be interpreted with caution. The pooled effect sizes should primarily be interpreted as broad statistical indications across heterogeneous contexts rather than definitive evidence regarding which specific interventions are most effective for particular populations, diagnoses, or outcomes. Consequently, conclusions regarding clinical effectiveness remain exploratory, and the findings cannot support strong intervention-specific recommendations across all paediatric swallowing and feeding populations. These findings have important implications for clinical practice, suggesting that intervention selection should be guided by specific functional targets rather than assuming generalised effects across feeding and swallowing domains. Overall, these results support domain-specific interventions, particularly for drooling and oral-motor function, in children with neurodevelopmental conditions. In particular, interventions that are developmentally appropriate and evaluated using comprehensive multi-item outcome measures appear most likely to demonstrate clinically meaningful effects. These findings highlight the importance of selecting outcome measures that capture functional improvements relevant to daily swallowing and feeding activities and indicate a need to re-examine current approaches to swallowing and feeding interventions. Evidence for swallowing and feeding remains limited, highlighting important gaps in the literature, raising concerns about the effectiveness of current interventions, and underscoring the need for further high-quality trials to inform clinical practice and optimise intervention strategies.

These findings should also be considered within the broader context of the ICF and PFD frameworks, as well as the integrative framework proposed in this manuscript, which conceptualises swallowing and feeding difficulties as multidimensional and distinguishes domain-specific patterns of intervention effectiveness. The predominance of outcomes focused on isolated behavioural or oral-motor components in the included studies highlights a limited alignment with these frameworks and with the domain structure applied in this review. Future research would benefit from explicitly integrating PFD and ICF perspectives when designing interventions and selecting outcome measures, and from adopting domain-specific frameworks such as the one applied in this review, to better capture the complexity of swallowing and feeding disorders and to ensure that clinically meaningful, domain-relevant outcomes are addressed.

### 4.3. Limitations

Although this review was conducted and reported in line with PRISMA guidelines to minimise potential bias and enhance methodological transparency, several limitations inherent to the study should be considered. In particular, substantial heterogeneity across the included studies—in terms of study design, participant characteristics, intervention protocols, and outcome measures—limited the scope and robustness of the meta-analyses. In addition, the relatively small number of RCTs within several domains limited the precision of pooled estimates and the reliability of subgroup analyses. A broader domain-level synthesis was therefore prioritised because the limited number of available RCTs within more homogeneous intervention subgroups restricted the feasibility and statistical reliability of more narrowly focused meta-analyses. This approach enabled the identification of broader patterns across the domains of swallowing and feeding interventions while acknowledging the resulting heterogeneity and limitations in clinical specificity and interpretability. This variability complicated direct comparisons between studies and reduced the feasibility of pooling data. Therefore, the overall conclusions, including the results of the meta-analyses, should be interpreted with appropriate caution.

## 5. Conclusions

This systematic review synthesised evidence from RCTs examining behavioural interventions and botulinum toxin injections for children with drooling, swallowing, and feeding difficulties, demonstrating that the effectiveness of interventions varies across functional domains. The findings indicate that these interventions can produce meaningful improvements in drooling and oral-motor function, with effect sizes in the moderate-to-large range. Intervention effects were influenced by factors such as age, intervention type, and the nature of the outcome measures, with stronger effects observed in older children for drooling and in younger children for mastication. However, given the substantial heterogeneity across study populations, interventions, and outcome measures, these findings should primarily be interpreted as exploratory indications of potential domain-specific effects rather than definitive evidence supporting specific clinical interventions or treatment pathways.

In contrast, evidence for feeding and swallowing outcomes remains limited and inconclusive. The absence of significant effects in these domains likely reflects both the small number of available trials and the complex, multidimensional nature of feeding and swallowing difficulties, which often involve interacting medical, nutritional, behavioural, and environmental factors. As a result, interventions focusing on isolated behavioural components may be insufficient to produce measurable improvements across these broader functional domains.

The review also highlights substantial heterogeneity across interventions, study populations, and outcome measures, which complicates comparisons across studies and limits the precision of pooled estimates. In particular, the use of diverse, sometimes non-standardised outcome measures limits the ability to synthesise findings across trials and underscores the need for psychometrically robust, standardised assessment tools in paediatric swallowing and feeding research. Consequently, conclusions regarding clinical effectiveness should be interpreted with caution, as pooled effect sizes across heterogeneous contexts remain largely exploratory and cannot support strong condition- or intervention-specific clinical recommendations.

Future research should prioritise well-designed, adequately powered RCTs, with clear reporting of intervention protocols, consistent use of validated outcome measures, and longer-term follow-up to evaluate sustained treatment effects. Greater alignment of outcome measures across studies would facilitate more robust meta-analyses and strengthen the paediatric evidence base. In addition, future work should consider the multidimensional nature of paediatric swallowing and feeding disorders, integrating behavioural, medical, and developmental perspectives to better inform clinical decision making.

Overall, the current evidence supports domain-specific use of behavioural interventions and botulinum toxin injections to improve drooling and oral-motor function in children, while identifying critical gaps in the literature regarding interventions for swallowing and feeding difficulties. Addressing these gaps through rigorous, domain-specific research will be essential to advancing evidence-based care and improving functional outcomes for children with swallowing and feeding disorders, particularly through well-designed, adequately powered RCTs that employ standardised, psychometrically robust outcome measures.

## Figures and Tables

**Table 1:** Literature databases and search strategies.

Database and Search Strategies
**CINAHL Ultimate**: ((MH “Deglutition”) OR (MH “Deglutition Disorders”) OR (MH “Feeding of Disabled”) OR (MH “Feeding and Eating Disorders of Childhood”) OR (MH “Infant Feeding”) OR (MH “Infant Feeding, Supplemental”) OR (MH “Parenteral Feeding (Saba CCC)”) OR (MH “Infant Feeding Pattern Impairment (Saba CCC)”) OR (MH “Ineffective Infant Feeding Pattern (NANDA)”) OR (MH “Feeding Self Care Deficit (NANDA)”) OR (MH “Feeding Methods”) OR (MH “Eating Behavior”) OR (MH “Eating Disorders Management (Iowa NIC)”) OR (MH “Eating Disorders”) OR (MH “Eating”) OR (MH “Sialorrhea”) OR (MH “Saliva”) OR (MH “Salivation”) OR (MH “Mastication”)) AND (MH “Randomized Controlled Trials”) *Limits*: Subject Age: all child.
**Embase**: (Swallowing/OR Dysphagia/OR Feeding/OR Eating/OR Eating disorders/OR hypersalivation/OR salivation/OR salivation disorder/OR saliva/OR mastication/) AND (randomization/OR randomized controlled trial/OR “randomized controlled trial (topic)”/OR controlled clinical trial/) *Limits*: child <unspecified age>
**PubMed**: (“Deglutition” [Mesh] OR “Deglutition Disorders” [Mesh] OR “Feeding and Eating Disorders of Childhood” [Mesh] OR “Feeding and Eating Disorders” [Mesh] OR “Feeding Behavior” [Mesh] OR “Sialorrhea” [Mesh] OR “Saliva” [Mesh] OR “Salivation” [Mesh] OR “Mastication” [Mesh]) AND (“Randomized Controlled Trial” [Publication Type] OR “Randomized Controlled Trials as Topic” [Mesh] OR “Controlled Clinical Trial” [Publication Type] OR “Pragmatic Clinical Trials as Topic” [Mesh]) *Limits*: Child: birth-18 years

**Table 2:** Mapping of outcome measures from included studies onto the conceptual framework.

Study	Drooling			Swallowing				Feeding				Oral-Motor Function	
	Drooling Severity	Drooling Frequency	Combined Severity/ Frequency	Swallowing Safety	Swallowing Efficiency	Combined/ Undefined	Health Outcomes	Consistency Management	Feeding Route/ Tube Dependency	Feeding Independence	Mealtime Efficiency	Oral-Motor Skills	Mastication
Abd-Elmonem et al. (2021) [23]												X	X
Acar et al. (2022) [24]								X		X			
Akaltun et al. (2023) [25]								X			X		
Alrefai et al. (2009) [26]	X	X	X										
Awan et al. (2017) [27]	X	X											
Basciani et al. (2011) [28]			X										
Bekkers et al. (2019) [29]	X												
Bekkers et al. (2021) [30]	X												
Berweck et al. (2021) [31]	X												
Fan et al. (2020) [32]			X									X	X
Gisel et al. (1995) [34]				X							X		
Gisel (1996) [33]				X							X		
Haberfellner et al. (2001) [35]				X					X				X
Inal et al. (2017) [36]	X	X										X	X
Korbmacher et al. (2004) [37]						X						X	
Mokhlesin et al. (2022) [38]			X										
Mokhlesin et al. (2024) [39]						X						X	
Nordgarden et al. (2012) [40]			X										
Pervez et al. (2022) [41]	X												
Reid et al. (2008) [42]			X										
Serel Arslan et al. (2017) [43]													X
Siğan et al. (2013) [44]			X	X		X				X		X	X
Song et al. (2015) [45]				X				X				X	X
Umay et al. (2020) [46]			X	X	X	X		X				X	X
Wilken et al. (2008) [47]	X												
Wu et al. (2011) [48]			X										
Yilmaz et al. (2024) [49]	X	X	X										
Zengin et al. (2025) [50]				X									

Note. Data highlighted in green were used in the meta-analysis.

**Table 3:** Between group meta-analyses comparing experimental and comparison groups across all four domains: drooling, swallowing, feeding, and oral-motor functions.

Group	*k*	Hedge’s *g*	Lower Limit CI	Upper Limit CI	*Z*-Value	*p*-Value
***Drooling***	10	0.629	0.062	1.195	2.175	0.030 *
***Swallowing***	4	−0.126	−0.533	0.281	−0.606	0.544
***Feeding***	4	0.002	−0.453	0.456	0.007	0.994
***Oral-motor functions***						
— Oral-motor skills	6	0.629	0.270	0.987	3.436	0.001 *
— Mastication	6	0.992	0.020	1.965	1.999	0.046 *

Notes. * Significant; (*k* = number of studies contributing to each subgroup analysis).

**Table 4:** Between-subgroup meta-analyses comparing intervention groups of included studies.

Subgroup	*k*	Hedge’s *g*	Lower Limit CI	Upper Limit CI	*Z*-Value	*p*-Value
***Drooling***						
Outcome measure						
— Multiple-item	5	1.416	0.228	2.605	2.336	0.010 *
— Single-item	5	0.163	−0.259	0.585	0.757	0.449
Respondent						
— Caregiver	3	2.404	0.302	4.506	2.241	0.025 *
— Clinician	5	0.369	0.057	0.681	2.320	0.020 *
— Teacher	2	−0.327	−0.867	0.212	−1.188	0.235
Diagnostic group						
— Cerebral palsy	6	0.605	−0.144	1.355	1.583	0.113
— Mixed population	4	0.657	−0.287	1.600	1.364	0.173
Age						
— 2–5 years	5	0.179	−0.140	0.499	1.102	0.271
— 6–11 years	5	1.284	0.122	2.446	2.166	0.030 *
Intervention type						
— Botulinum toxin	3	2.530	0.654	4.406	2.643	0.008 *
— Botulinum toxin vs. Botulinum toxin	2	0.200	−0.816	1.215	0.385	0.700
— Other	5	0.128	−0.170	0.427	0.842	0.400
***Swallowing***						
Outcome measure						
— Multiple-item	3	−0.249	−0.686	0.188	−1.118	0.264
— Single-item	1	0.353	−0.493	1.200	0.818	0.413
Diagnostic group						
— Cerebral palsy	3	−0.206	−0.816	0.405	−0.660	0.509
— Down syndrome	1	0.000	−0.623	0.623	0.000	1.000
***Feeding***						
Age						
— 2–5 years	2	0.275	−0.109	0.659	1.403	0.161
— 6–11 years	2	−0.419	−1.029	0.192	−1.344	0.179
***Oral-motor functions***						
**Oral-motor skills**						
Outcome measure						
— Multiple-item	1	1.034	0.136	1.93	2.256	0.024 *
— Single-item	3	0.552	0.161	0.943	2.765	0.006 *
Age						
— 2–5 years	2	0.604	0.145	1.063	2.581	0.010 *
— 6–11 years	2	0.674	0.004	1.345	1.972	0.049 *
**Mastication**						
RoB 2						
Low	5	0.672	−0.254	1.598	1.422	0.155
Some concerns	1	2.572	1.973	3.172	8.405	<0.001 *
Age						
— 2–5 years	4	1.519	0.420	2.619	2.708	0.007 *
— 6–11 years	2	−0.130	−0.734	0.475	−0.420	0.674

Notes. * Significant; (*k* = number of studies contributing to each subgroup analysis).

## Data Availability

No new data were created or analysed in this study. Data sharing is not applicable.

## Supplementary Materials

The following supporting information can be downloaded at: https://www.mdpi.com/article/10.3390/jcm15124653/s1, Table S1: PRISMA 2020 Abstract Checklist; Table S2: PRISMA 2020 Checklist; Table S3: Study characteristics of behavioural interventions and botulinum toxin injections for paediatric drooling, feeding, and swallowing problems; Table S4: Outcome of behavioural interventions and botulinum toxin injections for paediatric drooling, feeding and swallowing problems.
