# Supplementary material for: Behavioural Interventions and Botulinum Toxin Injections for Drooling, Swallowing, Feeding, and Oral-Motor Outcomes in Children: A Domain-Specific Systematic Review and Meta-Analysis of Randomised Controlled Trials
Source: J Clin Med. 2026 Jun 16;15(12):4653. doi: 10.3390/jcm15124653 (PMC13302669; doi:10.3390/jcm15124653)
Supplement: Supplementary file 1 [file jcm-15-04653-s001.zip › Supplementary Table S3.pdf]

Supplementary Table S3. Study characteristics of behavioural interventions and botulinum toxin injections for paediatric drooling, feeding, and swallowing problems.

**Study**<br>• **Country**	**Drooling, Swallowing, and Feeding problems**[a] (Definition/terminology; diagnostic measure/method)<br>**Diagnosis**<br>**Main inclusion/exclusion criteria**	**Sample (N)**<br>• **Groups (n)**[a]	**Group descriptive (Mean ± SD)**<br>(Age, gender, relevant medical diagnoses)
Abd-Elmonem et al., 2021 [1]<br>• Egypt	**Oral-motor skills:** NR<br>**Diagnosis:** Cerebral palsy (spastic quadriplegia)<br>**Inclusion:** Children aged between 12 and 48 months with a diagnosis of spastic quadriplegia, ≤10 on an initial evaluation of Oral-motor Assessment Scale and having a least a problem of oral-motor functions (drooling, swallowing and/or sucking) and independent feeding; grade ³ 2 spasticity according to the Modified Ashworth Scale, and at level IV and V motor function according to the Gross Motor Function Classification-Extended and Revised; partial head and trunk control<br>**Exclusion:** Gum and/or dental problems, congenital problems of the mouth and soft palate, uncontrolled seizures, metabolic disorders, cardiopulmonary disorders, significant mental problems	N=64<br>- Experimental group: Oral sensorimotor stimulation and neurodevelopmental training-based sequenced trunk co-activation exercises (n=32)<br>- Control group: Neurodevelopmental training-based sequenced trunk co-` exercises (n=32)	Experimental group / Control group<br>**Age:** 29.7±8.09mo / 29.2±7.97mo<br>**Male:** 47% / 47%<br>**Median GMFCS Level (IQR):** 4 (5.4) / 4 (5.4)<br>**Weight:** 10.0±1.04 kg / 10.0±1.23 kg<br>**Gross motor function measured using Gross Motor Function Measure (GMFM-88):** 21.37±2.09 / 20.94±2.51
Acar et al., 2022 [2]<br>• Turkey	**Feeding difficulties/disturbances:** lack of food retention, poor food mastication, delayed swallowing, breathing/swallowing incoordination, airway interference, and a predisposition to aspiration; identified using a four-item questionnaire about mealtime length, mealtime stress, respiratory symptoms, and weight gain (Arvedson, 2013) [3]<br>**Diagnosis:** Cerebral palsy	N=40<br>- Experimental group: Neck and trunk stabilisation exercise added to feeding and oral-motor interventions (n=20)<br>- Control group: Feeding and oral-motor intervention only (n=20)	Experimental group / Control group<br>**Age:** 3.20±0.76y / 2.97±0.91y<br>**Male:** 40% / 55%<br>**Height:** 91.90±8.50cm / 91.50±10.27cm<br>**Weight:** 11.25±2.40 / 11.74±2.8cm<br>**BMI:** 13.27±1.89 kg/m² / 14.02±3.74kg/m²<br>**Preterm birth:** 60% / 40%<br>**Subtypes of cerebral palsy:**

	**Inclusion:** At least two positive responses on Arvedson (2013) [3]<br>**Exclusion:** NR		**Diplegic:** 15% / 5%<br>**Hemiplegic:** 10% / 10%<br>**Quadriplegic:** 40% / 50%<br>**Dyskinetic:** 5% / 10%<br>**Hypotonic:** 30% / 25%<br>**GMFCS level:**<br>**Level 1:** 20% / 10%<br>**Level 3:** 10% / 0%<br>**Level 4:** 20% / 20%<br>**Level 5:** 50% / 70%<br>**Mini-Manual Ability Classification System**<br>**Level 1:** 5% / 5%<br>**Level 2:** 20% / 15%<br>**Level 3:** 25% / 25%<br>**Level 4:** 15% / 10%<br>**Level 5:** 35% / 45%<br>**Eating and Drinking Ability Classification System** (n=16 / n=11)<br>**Level 1:** 6.25% / 0%<br>**Level 2:** 18.75% / 9%<br>**Level 3:** 31.25% / 63%<br>**Level 4:** 31.25% / 18%<br>**Level 5:** 12.5% / 9%
Akaltun et al., 2023 [4]<br>• Turkey	**Dysphagia:** NR<br>**Diagnosis:** Cerebral palsy<br>**Inclusion:** Children with cerebral palsy who had oro-pharyngeal dysphagia symptoms or findings and were subsequently hospitalised and rehabilitated.	N=101<br>- Experimental group: Kinesio tape (n=54)<br>- Control group: Sham (n=47)	Experimental group / Control group<br>**Age:** 50.4±17.4mo / 47.9±18.6mo<br>**Male:** 63% / 53.2%<br>**Height:** 94.6±15.8cm / 91.3±17.9cm<br>**Weight:** 15.0±5.0kg / 14.5±8.4kg<br>**History:**

	**Exclusion:** A history of maxillary, head, or neck surgery or botulinum toxin injection, structural oropharyngeal abnormality, known oesophageal dysphagia or gastroesophageal reflux disease, who received medical or physical therapy for dysphagia in the last six months, using drugs for seizures or spasticity, and tube-dependency with no oral intake		**Prematurity:** 53.7% / 48.9%<br>**Multiple pregnancy:** 22.2% / 21.3%<br>**Birth trauma:** 5.6% / 8.6%<br>**Infection:** 7.4% / 10.6%<br>**Maternal predisposing factor:** 11.1% / 10.6%<br>**Consanguineous marriage:** 14.8% / 23.4%<br>**Additional problems:**<br>**Mental retardation:** 61.1% / 57.5%<br>**History of epilepsy:** 64.8% / 44.7%<br>**Hearing disorder:** 9.3% / 17.0%<br>**Vision disorder:** 40.7% / 38.3%<br>**Speech disorder:** 59.3% / 61.7%<br>**Bowel incontinence:** 81.5% / 80.9%<br>**Dental disorder:** 79.6% / 72.3%<br>**GMFCS (1-5):** 4.1±1.1 / 3.9±1.1<br>**Motor limb distribution:**<br>**Hemiplegia:** 13.0% / 19.1%<br>**Diplegia:** 24.0% / 23.4%<br>**Triplegia:** 44.5% / 44.7%<br>**Tetraplegia:** 18.5% / 12.8%<br>**Presence of symptoms/findings:**<br>**Drooling:** 75.9% / 72.3%<br>**Poor lip movements:** 72.2% / 66.0%<br>**Poor tongue movements:** 87.1% / 80.9%<br>**Difficulty biting:** 46.3% / 44.7%<br>**Difficulty chewing:** 88.9% / 83.0%<br>**Difficulty drinking:** 14.8% / 10.6%<br>**Coughing/choking during/after feeding:** 51.9% / 40.2%

			**Retching/vomiting during/after feeding:** 33.3% / 25.5%<br>**Reduced in pulse O_2 saturation:** 25.9% / 25.5%<br>**Functional Oral Intake Scale (1-7):** 5.1±1.2 / 5.4±0.9<br>**Mealtime:** 47.0±11.3 minutes / 44.1±13.3 minutes
Alrefai et al., 2009 [5]<br>• Jordan	**Sialorrhea:** NR<br>**Diagnosis:** Cerebral palsy<br>**Inclusion:** Children who scored seven or higher on a rating scale of drooling frequency and severity (Thomas Stonells drooling scale?), screened by the physician at a multidisciplinary cerebral palsy rehabilitation centre<br>**Exclusion:** Children who received oral treatment for drooling in the last three months or had received botulinum toxin injection for any other indication in the last 6 months	N=24<br>- Experimental group: Botulinum toxin injection (n=11)<br>- Control group: Placebo (normal saline solution) (n=13)<br><br>12 of N=24 were followed up after the second injection, four months after the initial injection<br>- Experimental group: Botulinum toxin injection (n=7)<br>- Control group: Placebo (normal saline solution) (n=5)	Experimental group / Control group<br>**Age:** 3.5±1.7y / 4.5±2.0y<br>**Male:** 63.6% / 61.5%<br>**Frequency of drooling:**<br>**Median:** 4 / 4 (constantly drools)<br>**Constantly drools:** 36.4% / 38.5%<br>**Frequently drools:** 63.6% / 61.5%<br>**Severity of drooling:**<br>**Median:** 5 / 5 (profuse)<br>**Profuse:** 18.2% / 0%<br>**Severe:** 81.8% / 100%<br>**Median total drooling score:** 9 / 9
Awan et al., 2017 [6]<br>• Iran	**Drooling:** NR<br>**Diagnosis:** Cerebral palsy<br>**Inclusion:** Aged between 4 and 8 years; Drooling rate of ≥ 3 on Thomas Stonells drooling scale; Good understanding of verbal commands; Good head control<br>**Exclusion:** Undergoing any other treatment strategy for drooling, including medications, recent surgery, history of intra-salivary gland injections, uncontrolled seizures, uncorrected or corrected cleft palate or cleft lip and open wounds around lips or on face	N=48<br>- Experimental group 1: Kinesio taping and oral-motor exercises (n=24)<br>- Experimental group 2: Kinesio taping only (n=24)	Experimental group 1 / Experimental group 2<br>**Age:** 3.93±1.26y / 3.66±1.26<br>**Male:** 62.5% / 58.3%<br>**Type of cerebral palsy:**<br>**Spastic:** 16.7% / 16.7%<br>**Dyskinetic:** 41.7% / 58.3%<br>**Ataxic:** 8.3% / 8.3%<br>**Flaccid:** 16.7% / 4.2%<br>**Mixed:** 12.5% / 16.7%<br>**GMFCS:**<br>**Level III:** 12.5% / 12.5%<br>**Level IV:** 37.5% / 33.3%

			**Level V:** 45.8% / 58.3%
Basciani et al., 2011 [7]<br>• Italy	**Sialorrhea:** NR<br>**Diagnosis:** Cerebral palsy<br>**Inclusion:** Children with refractory sialorrhea or drooling. Sialorrhea was considered refractory when all common therapeutic agents, including anticholinergic drugs, failed.<br>**Exclusion:** A history of any surgical procedure to the head and neck to reduce salivation, use of any medications for sialorrhea, and use of any pharmacological agents that could affect salivary production	N=27<br>- Experimental group 1: Low-dose Botulinum toxin B injection (n=6, 1 lost to follow-up at 12 weeks)<br>- Experimental group 2: Medium dose Botulinum toxin B injection (n=7, 1 lost to follow-up at 12 weeks)<br>- Experimental group 3: High dose Botulinum toxin B injection (n=7, none lost to follow-up)<br>- Control group: No treatment (n=7, 1 lost to follow-up at 12 weeks)	Low dose / Medium dose / High dose / Control<br>**Age:** 8.3±1.3y / 7.6±1.6y / 7.6±1.8y / 7.6±1.4y<br>**Male:** 66.7% / 57.1% / 42.9% / 57.1%<br>**Weight:** 24.5±4.2kg / 25.2±9.6kg / 23.5±8.1kg / 22.8±4.0kg<br>All children had severe neurological dysfunction consisting of mixed disorders, including spastic paraparesis, tetraparesis, dystonic movements and ataxia. GMFCS levels ranged from III to V. All children had moderate to severe intellectual disability, and 22.2% had epilepsy.
Bekkers et al., 2019 [8]<br>• The Netherlands	**Drooling:** NR<br>**Diagnosis:** nonprogressive neurodevelopmental disabilities (majority CP)<br>**Inclusion:** Severe drooling (Drooling Frequency scale score ³ 3 or Drooling Severity scale score ³ 2), aged 8 years and older; cerebral palsy or any other nonprogressive neurodevelopmental disability; ability and willingness to follow the study protocol and attend the 8- and 32-week visits; written and informed consent from caregivers, and when appropriate, oral consent from the child<br>**Exclusion:** Progressive oral-motor impairment, already receiving medical treatment (glycopyrrolate or scopolamine), a surgical history involving submandibular duct ligation, any other contraindication for general anaesthesia, Botulinum toxin A injections, or surgery, or concurrent use of benzodiazepines	N=53<br>- Experimental group 1: Botulinum toxin type A injection (n=26)<br>- Experimental group 2: Submandibular duct ligation (n=27)	Experimental group 1 / Experimental group 2<br>**Age:** 11.2±2.5y / 11.1±3.2y<br>**Male:** 57.7% / 59.3%<br>**Main diagnosis:**<br>**Spastic CP:** 38.5% / 22.2%<br>**Dyskinetic CP:** 3.8% / 11.1%<br>**Spastic/dyskinetic CP:** 19.2% / 18.5%<br>**CP, unknown type:** 3.8% / 0%<br>**Other neurodevelopmental disability:** 34.6% / 48.1%<br>**GMFCS level (CP only)**<br>**II:** 11.8% / 7.1%<br>**III:** 17.6% / 0%<br>**IV:** 29.4% / 57.1%<br>**V:** 41.2% / 35.7%<br>**Degree of disability**<br>**Ambulant:** 42.3% / 37.0%<br>**Non-ambulant:** 57.5% / 63.0%

			**Developmental age:**<br>**<4y:** 57.7% / 55.6%<br>**>4y:** 42.3% / 44.4%<br>**Epilepsy:**<br>**Yes:** 65.4% / 55.6%<br>**Controlled:** 76.5% / 86.7%<br>**Intractable:** 23.5% / 13.3%<br>**No:** 34.6% / 44.4%<br>**Gastroesophageal reflux disease:** 30.8% / 33.3%<br>**Dental malocclusion:**<br>**Normal:** 36.0% / 26.9%<br>**Mild:** 32.0% / 50.0%<br>**Severe:** 32.0% / 23.1%<br>**Missing (n):** 1 / 1<br>**Mouth closure:**<br>**Normal:** 3.8% / 0%<br>**Incomplete:** 34.6% / 26.9%<br>**Constantly open:** 61.5% / 73.1%<br>**Missing (n):** 0 / 1<br>**Gastrostomy feeding:**<br>**Oral:** 61.5% / 74.1%<br>**Gastrostomy/gastrostomy and oral (no pharyngeal swallowing problem):** 38.5% / 25.9%<br>**Botulinum toxin A pretrial:** 57.7% / 63.0%<br>**Number of Botulinum toxin A injections received:** 1.6±1.8 / 1.4±1.3<br>**Time since last injection:** 1.1y±0.7y / 2.0±2.8y
Bekkers et al., 2021 [9]	Same sample as Bekkers et al. (2019) [8]	Same conditions/groups as Bekkers et al. (2019) [8]	Same sample as Bekkers et al. (2019) [8]

• The Netherlands			
Berweck et al., 2021 [10]<br>• Germany	**Sialorrhea (drooling):** NR<br>**Diagnosis:** Neurologic disorders or intellectual disability<br>**Inclusion:** Children and adolescents (aged 2 to 17 years) who had a neurologic disorder (e.g. CP or traumatic brain injury) or intellectual disability associated with chronic sialorrhea for ^{3} 3 months before screening; a modified teacher's drooling scale score of ^{3} 6 ("severe drooling to the extent that clothing becomes damp occasionally") rated by the investigator<br>**Exclusion:** Sialorrhea not related to neurologic disorders or intellectual disability; body weight < 12kg; clinically present moderate or severe dysphagia (choking more than once a week), except for patients on parenteral nutrition or nutrition via gastrostomy; paediatric epilepsy not well controlled with antiepileptic drugs; any previous treatment with botulinum toxin for any body region during the year before screening or within the screening period; extremely poor dental/oral condition	N=218 (Only the main period findings from children and adolescents aged 6-17 years)<br>- Experimental group: Incobotulinumtoxin A (n=146)<br>- Control group: Placebo (n=70)<br><br>n=129 in the Experimental group and n=60 in the Control group also participated in the extension period<br><br>No randomisation for age group 2-5 (considered unethical)	Experimental group (n=148) / Control Group (n=72)<br>**Age:** 10.4±3.17 y / 10.3±325 y<br>**Male:** 62.8% / 62.5%<br>**BMI:** 15.8±3.25 / 16.4±3.65<br>**Intellectual disability:** 87.8% / 88.9%<br>**Primary diagnosis leading to sialorrhea:**<br>**CP:** 68.9% / 59.7%<br>**Traumatic brain injury:** 6.1% / 1.4%<br>**Other:** 25.0% / 38.9%<br>**Baseline assessment:**<br>**Unstimulated salivary flow rate:** 0.570.25g/min 0.6±0.25g/min<br>**Carers' modified Teacher's Drooling Rating:** 7.6±1.2 / 7.7±1.2<br>**Investigators' modified Teacher's Drooling Rating:** 7.7±1.1 / 7.7±1.2<br>**Drooling Quotient:** 42.45±22.32 / 46.81±25.25<br>**GMFCS (expanded and revised) for CP only**<br>**I:** 4.9% / 7.0%<br>**II:** 34.3% / 25.6%<br>**III:** 10.8% / 25.6%<br>**IV:** 19.6% / 25.6%<br>**V:** 30.4% / 16.3%
Fan et al., 2020 [11]<br>• China	**Oral-motor dysfunction:** Includes chewing dysfunction, drooling and strong tongue thrust.<br>**Diagnosis:** Cerebral palsy	N=48<br>- Experimental group 1: Oral-motor training (n=24)<br>- Experimental group 2: Functional chewing training (n=24)	Experimental group 1 / Experimental group 2<br>**Age**: between 4 and 6 years for both groups<br>**Male**: 67% / 54%<br>**Gross Motor Function Classification System Level**:

	**Inclusion:** Aged between 4 and 6 years, cerebral palsy, oral-motor dysfunction, able to feed orally and have an intellectual level capable of undergoing training<br>**Exclusion:** Younger than 4 years and older than 6 years of age, severe other disease that prevents training, previous drug therapy or surgical treatment affecting swallowing		**Level 1**: 4% / 4%<br>**Level 2:** 17% / 12%<br>**Level 3:** 21% / 25%<br>**Level 4:** 8% / 8%<br>**Level 5:** 50% / 50%<br>**Oral-motor assessment:**<br>**Inability to close mouth:** 83% / 92%<br>**Open bite:** 38% / 62%<br>**High-arched palate**: 58% / 46%<br>**Gag reflex:** 75% / 88%<br>**Karaduman Chewing Performance Scale level**<br>**Level 0:** 0% / 0%<br>**Level 1**: 8% / 4%<br>**Level 2:** 13% / 8%<br>**Level 3:** 38% / 42%<br>**Level 4:** 42% / 46%<br>**Tongue Thrust Rating Scale level**<br>**Level 0:** 0% / 0%<br>**Level 1**: 4% / 8%<br>**Level 2**: 33% / 25%<br>**Level 3:** 63% / 67&
Gisel et al., 1995 [12]<br>• Canada	**Aspiration:** Defined to indicate penetration and/or aspiration for convenience; where penetration describes the flow of food into the airway immediately before or during the apnoeic period of swallowing, and aspiration describes when food enters the airway following a swallow, assisted by inhalation during the resumption of respiration	N=27<br>- Experimental group: Sensorimotor treatment for children who did not aspirate (20 weeks) (n=10)<br>- Control group 1: 10 weeks of the school feeding routine for 10 weeks (no treatment) followed by 10 weeks of sensorimotor treatment for children who did not aspirate (n=10)	Experimental / Control 1 / Control 2<br>**Age:** 4.8±1.4y / 5;0±1.9y / 5.4±2.7y<br>**Male:** 30.0% / 40.0% / 100%<br>All children had a diagnosis of cerebral palsy with moderate to severe impairment<br>Children's weight was at the 5th percentile for their age and at or below the 35th percentile for skinfold measures

	**Diagnosis:** Cerebral palsy (who had moderate eating impairments)<br>**Inclusion:** Could eat a solid texture and a puree, within one and two standard deviations of the established time norms, respectively.<br>**Exclusion:** NR	- Control group 2: 10 weeks of the school feeding routine for 10 weeks (no treatment) followed by 10 weeks of sensorimotor treatment for children who aspirated (n=7)	Nineteen children used wheelchairs; two children used walkers, and six could walk<br>All children required assistance with activities of daily living and had hypo- to hypertonicity in their trunk and extremities<br>The severity of spasticity varied between upper and lower extremities and between the right and left body sides, although most were quadriparesis
Gisel, 1996 [13]<br>• Canada	**Eating impairment:** NR<br>**Diagnosis:** Cerebral palsy<br>**Inclusion:** A diagnosis of cerebral palsy with moderate-to-severe motor impairment; able to eat a standard solid texture within 1 standard deviation and a puree at or below 2 standard deviations of established time norms.<br>**Exclusion:** NR	N=34<br>- Experimental group: Sensorimotor treatment (n=11)<br>- Control group 1: Chewing only treatment (n=12)<br>- Control group 2: School feeding routine followed by sensorimotor treatment (n=12)	Experimental (n=11) / Control 1 (n=12) / Control 2 (n=12)<br>**Age:** 6.3±1.4y / 7.3±2.1y / 7.7±2.7y<br>**Male:** 45.5% / 66.7% / 50.0%<br>Children's weight was at the 5th percentile for their age and at or below the 35th percentile for skinfold measures<br>Twenty-seven children used wheelchairs; five were ambulatory; three used tricycles for ambulation<br>All children required assistance with activities of daily living and had hypo- to hypertonicity in their trunk and extremities<br>The severity of spasticity varied between upper and lower extremities and between the right and left body sides, although most were quadriparesis
Habefellner et al., 2001 [14]<br>• Canada	**Eating impairment:** NR<br>**Diagnosis:** Cerebral palsy<br>**Inclusion:** NR<br>**Exclusion:** NR	N=20<br>- Experimental group: Intraoral appliance (Innsbruck Sensorimotor Activator and Regulator) (n=10)<br>- Control group: Standard rehabilitation at school (n=10)	All children<br>**Age:** 8.3±0.9y<br>**Male:** 45.0%<br>**Dentition:**<br>**Primary dentition:** 40%<br>**Early mixed dentition:** 35%<br>**Late mixed dentition:** 10%

			**Permanent dentition:** 15%<br>All children had a diagnosis of cerebral palsy with tetraparesis and moderate motor impairment; ate a solid texture within 1 standard deviation of the norm of the Gisel video assessment but were 2 standard deviations above the norm for puree; were 1.5 standard deviations below standards in weight for age, and skinfolds were below the 50th centile for age; and were able to breathe through their noise<br>Fourteen children used wheelchairs exclusively; four used wheelchairs for long-distance transport but began to use assistive devices (tricycles, walkers, hand-hold for ambulation indoors); two were ambulatory<br>Eleven children were fully dependent in their activities of daily living; nine required partial assistance<br>Fourteen wore diapers regularly; children could indicate when they needed to go to the bathroom<br>Children varied in their communicative development, from no to severe impairment (based on questions that required a yes or no answer)<br>Ten children were on seizure medication
Inal et al., 2017 [15]<br>• Turkey	**Tongue thrust:** An oral reflex associated with sucking behaviour during infancy; **Drooling:** NR<br>**Diagnosis:** Cerebral palsy<br>**Inclusion:** Children with cerebral palsy referred to the clinic for tongue thrust and drooling, aged between 4 and 6 years, and fed orally.	N=32<br>- Experimental group: Functional Chewing Training (n=16)<br>- Control group: Classical oral-motor exercise (n=16)	Experimental group / Control group<br>**Age:** 60.43±9.01mo / 56.06±9.91mo<br>**Male:** 56.2% / 62.5%<br>**Age of diagnosis:** 5.9±8.8mo / 4.9±4.5mo<br>**First teething time:** 6.6±9.3mo / 9.0±11.1mo<br>**GMFCS level:**<br>**Level I:** 0% / 0%<br>**Level II:** 6.3% / 6.3%

	**Exclusion:** Children who were below the age of 4 years and used any medicine and/or oral appliances that could affect tongue thrust behaviour		**Level III:** 25.5% / 37.5%<br>**Level IV:** 0% / 0%<br>**Level V:** 68.8% / 56.3%<br>**Oral-motor assessment:**<br>**Open mouth:** 87.5% / 87.5%<br>**Open bite:** 56.3% / 31.3%<br>**High palate:** 50% / 68.8%<br>**Gag reflex:** 87.5% / 75.0%<br>**Bottle-feeding usage time:**<br>**0-12 months:** 36.4% / 7.1%<br>**12-24 months:** 6.3% / 6.3%<br>**24-36 months:** 6.3% / 35.7%<br>**36 months and over:** 45.5% / 50.0%
Korbmacher et al., 2004 [16]<br>• Germany	**Orofacial dysfunction:** Includes a visceral swallowing pattern or habitual mouth breathing and inhibits the physiological development of the jaw and dentition<br>**Diagnosis:** Orofacial dysfunction<br>**Inclusion:** Multiple orofacial dysfunctions and further ongoing treatments (orthodontic therapy, ergotherapy, physiotherapy)<br>**Exclusion:** NR	N= 45<br>- Experiential group: Face Former therapy (n=26 at T0, n=19 at T1, n=17 at T2)<br>- Control group: Conventional myofunctional therapy (n=19 at T0, n=14 at T1, n= 13 at T2)	Experimental group / Control group (at T0)<br>**Age years:** 8.3±3.1y / 8.4 ±2.9y<br>**Male:** 65.4% / 78.9%
Mokhlesin et al. 2022 [17]<br>• Iran	**Drooling:** Contra-volitional loss of saliva and other oral contents, which is considered unusual during wakefulness after 4 years of age<br>**Diagnosis:** Intellectual disability (cerebral palsy, microcephalic, Down syndrome, idiopathic)<br>**Inclusion:** Intellectual disability, aged 4-18 years, had drooling, could understand and follow simple instructions	N=18<br>- Experimental group: Oral-motor treatment and Kinesio Taping (n=9)<br>- Control group: Oral-motor treatment and sham treatment (n=9)	Experimental group / Control group<br>**Age:** 117.33±55.92mo / 124.33±36.59mo<br>**Male:** 67% / 45%<br>**Aetiology:**<br>**Cerebral palsy:** 45% / 33%<br>**Microcephalic:** 0% / 12%<br>**Down syndrome:** 22% / 0%<br>**Idiopathic:** 33% / 55%

	**Exclusion:** Poor head control, progressive neurological problems, seizures, vast dental decay and teething, history of Botulinum injection and surgical or medical therapy for breathing, radiotherapy of head and neck, missing more than three therapeutic sessions in the study, and allergic reaction to Kinesio Taping		
Mokhlesin et al. 2024 [18]<br>• Iran	**Swallowing disorder:** NR<br>**Diagnosis:** Cerebral palsy<br>**Inclusion:** A diagnosis of spastic cerebral palsy and impairments in the oral phase of swallowing, ages between 6 and 12 years<br>**Exclusion:** Progressive neurological disorders, severe visual or hearing impairments, intellectual disability, severe dental or oral structural problems, uncontrolled seizures, high risk of aspiration/pharyngeal phase problems	N=20<br>- Experimental group: Action observation training and sensorimotor therapy (n=10)<br>- Control group: Sham and sensorimotor therapy (n=10)	Experimental group / Control group<br>**Age years:** 8.6±2 y / 7.6±1.2 y<br>**Weight:** 19.5±3.8 kg / 15.4±2 kg<br>**Male:** 70% / 80%<br>**GERD:** 20% / 10%<br>**Sialorrhea:** 20% / 30%<br>**GMFCS level:**<br>**Level III:** 50% / 60%<br>**Level IV**: 50% / 60%<br>**EDACS**<br>**Level 2:** 40% /30%<br>**Level 3:** 60% / 70%
Nordgarden et al., 2012 [19]<br>• Norway	**Drooling:** Involuntary loss of saliva from the mouth<br>**Diagnosis:** Cerebral palsy<br>**Inclusion:** A diagnosis of cerebral palsy, aged between 6 and 18 years and severe drooling (drooling that is observed to occur several times per day, and with a drooling coefficient of 50% or more)<br>**Exclusion:** Body weight <15 kg, involvement in other medical studies, taking medication that affects saliva production in the 3 months prior to the study, contraindications for general anaesthesia, any treatment with botulinum toxin in the previous 6 months,	N=6<br>- Experimental group 1: Botulinum toxin A injection to both the parotid and submandibular salivary glands (n=5)<br>- Experimental group 2: Botulinum toxin A injection to the submandibular glands only (n=1)	All children<br>**Age:** 13.7y<br>**Male:** 33.3%

	concurrent use of muscle relaxant medication or vancomycin and pregnancy.		
Pervez et al., 2022 [20]<br>• Pakistan	**Drooling:** Unintentional loss of saliva from the mouth that usually occurs due to weakness of orofacial and palate-lingual musculature, mostly associated with neurodegenerative disorders<br>**Diagnosis:** Non-degenerative neurological disabilities<br>**Inclusion:** A diagnosis of non-degenerative neurological disabilities (cerebral palsy, traumatic brain injury, childhood stroke); having oral phase dysphagia; age between 2.5 and 11 years, a drooling severity rating of ≥4 on the Modified Teacher's Drooling Scale, able to comprehend simple verbal commands, 3-word sentence level speech and good head control.<br>**Exclusion:** Structural abnormality of the respiratory system, a corrected or uncorrected cleft palate or cleft lip, recent surgery or taking any medication or on any treatment to control drooling	N=20<br>- Experimental group 1: Kinesio taping (n=10)<br>- Experimental group 2: Manipulation therapy (n=10)	All children<br>**Age:** 5.4y<br>**Male:** 70%<br>**Diagnosis:**<br>Cerebral palsy: 90%<br>Childhood stroke: 10%
Reid et al., 2008 [21]<br>• Australia	**Drooling:** NR<br>**Diagnosis:** Cerebral palsy, intellectual disability (IQ <70), or other neurological disorders<br>**Inclusion:** children aged between 6 and 18 years, a significant problem with drooling, parents/carers able to understand the study requirements and give consent<br>**Exclusion:** previous botulinum toxin A injections to the salivary glands, previous saliva control surgery, any botulinum toxin A treatment in the previous 6 months, unfit for general anaesthesia, unwillingness to withhold	N=48:<br>- Experimental group: (n=24)<br>- Control group: no treatment (n=24)	Experimental group / Control group<br>**Age years:** 11.9 ± 3.2y / 11.11 ± 3.4y<br>**Male**: 70.8 / 41.6<br>**Main diagnosis**:<br>**Cerebral palsy**: 54.2% /7 5%<br>**Intellectual disability**: 37.5% / 25%<br>**Other**: 8.3% / 0% **Epilepsy**: 50% / 29.2%<br>**Mobility**:<br>**Walks independently:** 45.8% / 33.3 %<br>**Walks with aids:** 4.2% / 4.2 %<br>**Non-ambulant:** 50% / 62.5%

	oral anticholinergic medication for the length of the study, family history of poor compliance		**Communication:**<br>**No speech problem:** 8.4% / 4.2%<br>**Some motor problems:** 25% / 33.3%<br>**Little or no speech:** 66.7% / 62.5%<br>**Intellectual function (IQ):**<br>**No impairment (>70):** 20.8% / 12.5%<br>**Mild impairment (50-69):** 16.7% / 20.8%<br>**Moderate impairment (30-49):** 37.5% / 33.3% /<br>**Severe impairment (<30):** 25% / 33.3% 2<br>**Non-oral feeding:** 25% / 20.8%<br>**On drooling medication before study:** 33.3% / 33.3%
Serel Arslan et al., 2017 [22]<br>• Turkey	**Chewing:** Rhythmic oral-motor activity to comminute and soften solid food (Foster et al.,[23] 2013)<br>**Diagnosis:** Cerebral Palsy<br>**Inclusion:** A diagnosis of cerebral palsy; had complaints about chewing function and could not manage solid food; aged 18 months or older<br>**Exclusion:** Using any medicine<br>and/or oral appliances that could affect chewing performance	N=80<br>- Experimental group: Functional chewing therapy (n=50)<br>- Control group: Traditional oral-motor exercise (n=30)	Experimental group / Control group<br>**Age years:** 3.5±1.9y / 3.4±2.3y<br>**Male:** 62.0% / 53.3%<br>**Height in cm:** 89.83±12.85 / 85.70±14.12<br>**Weight in kg:** 12.91±4.15 / 12.04±3.40<br>**Mealtime (min):** 31.52±30.07 / 44.14±39.21<br>**Number of teeth:** 19.73±1.11 / 19.60±1.29<br>**Oral-motor assessment parameters**<br>Open mouth: 38%/50%<br>Open bite: 24% / 36.7%<br>Tongue trust: 36%/ 50%<br>High palate: 52% / 60%<br>Oral hygiene problems: 76% /83.3%<br>Gag reflex: 98% / 93.3%
Siğan et al., 2013 [24]<br>• Turkey	**Feeding problems:** Having at least one or more problems of oral-motor functions such as sucking, chewing, swallowing, drooling and independent feeding.<br>**Diagnosis:** Cerebral palsy	N=81<br>- Experimental group: Oral-motor therapy (n=41)<br>- Control group: Routine physiotherapy (n=40)	Experimental group / Control group<br>**Age:** 24.32±10.86mo / 28.15±10.22mo<br>**Male:** 37.5% / 50%<br>**Cerebral palsy type:**

	**Inclusion:** Aged between 12 and 42 months, diagnosis of cerebral palsy, having at least one or more problems of oral-motor functions such as sucking, chewing, swallowing, drooling and independent feeding<br>**Exclusion:** Non-participation for more than three sessions; not participating in a routine physiotherapy for the control group		**Tetraparesis:** 42.5% / 40.0%<br>**Diparesis:** 40.0% / 30.0%<br>**Hemiparesis:** 7.5% / 22.5%<br>**Hypotonia:** 10.0% / 5.0%<br>**Ataxic:** 0% / 2.5%<br>**Functional feeding assessment subscale:**<br>**Spoon feeding:** 76.48±25.73 / 79.30±27.85<br>**Biting:** 83.46±22.17 / 83.45±24.42<br>**Chewing:** 57.35±29.89 / 66.33±23.60<br>**Drinking:** 91.28±10.68 / 91.61±14.03<br>**Swallowing:** 79.21±18.38 / 74.73±23.48<br>**Bayley Scales of Infant Development-II:**<br>**Mental scale:** 9.97±7.82 / 14.3±9.85<br>**Motor scale:** 6.77±5.32 / 9.95±6.80
Song et al., 2015 [25]<br>• South Korea	**Dysphagia:** NR<br>**Diagnosis:** Cerebral palsy<br>**Inclusion:** Cerebral palsy, dysphagia confirmed by video fluoroscopy swallowing study (VFSS) or rehabilitation doctor<br>**Exclusion:** Disorder in vision or hearing, seizure disorders, pacemaker	N=20<br>- Experimental group: Oral sensorimotor treatment and neuromuscular electrical stimulation (n = 10)<br>- Control group: Oral sensorimotor treatment and sham treatment (n = 10)	Experimental group / Control group<br>**Age years:** 6.2±7.78 y / 6.0±2.40y<br>**Male:** 70% / 60%<br>**Type of cerebral palsy**:<br>**Hemiplegia:** 20% / 40%<br>**Diplegia:** 50% / 30%<br>**Quadriplegia:** 30% / 20%<br>**Flaccid:** 0% / 10%
Umay et al., 2020 [26]<br>• Turkey	**Dysphagia:** NR<br>**Diagnosis:** Cerebral palsy<br>**Inclusion:** Cerebral palsy and any oropharyngeal dysphagia symptoms and/or findings, aged between 2 and 6 years<br>**Exclusion:** History of maxillary, head or neck surgery	N=102<br>- Experimental group: Conventional dysphagia rehabilitation and intermittent galvanic stimulation (n=52)<br>- Control group: Conventional dysphagia rehabilitation and sham stimulation (n=50)	Experimental group / Control group<br>**Age:** 51.97±24.46mo / 47.95±23.18mo<br>**Male:** 55.8% / 46.0%<br>**Height:** 101.87±16.04cm / 94.03±15.83cm<br>**Weight:** 15.01±5.23kg / 14.63±7.56kg<br>**Type of cerebral palsy:**<br>**Spastic:** 94.2% / 94.0%

	or botulinum toxin treatment, structural oropharyngeal abnormality, esophageal dysphagia and/or gastroesophageal reflux disease, medical and/or physical therapy for dysphagia, severe cognitive, visual, auditory, and sensory impairments, drug use due to seizure or spasticity, serious pulmonary or cardiac disease, or bleeding risk		**Dyskinetic:** 3.9% / 6.0%<br>**Hypotonic/Ataxic:** 1.9% / 0%<br>**Motor limb distribution:**<br>**Hemiplegia:** 32.7% / 36.0%<br>**Diplegia:** 13.5% / 14.0%<br>**Triplegia/quadriplegia**: 53.8% / 50.0%<br>**GMFCS level:**<br>**Level I:** 0% / 0%<br>**Level II:** 13.5% / 22.0%<br>**Level III:** 19.2% / 22.0%<br>**Level IV:** 42.3% / 32.0%<br>**Level V:** 25.0% / 24.0%
Wilken et al., 2008 [27]<br>• Germany	**Drooling:** NR<br>**Diagnosis:** Neurological disorders, including cerebral palsy, neuromuscular diseases, cortical malformations, neurometabolic and neurodegenerative disorders<br>**Inclusion:** A drooling score >3 on the Teacher's Drooling Scale	N=30<br>- Experimental group 1: Botulinum toxin A injection (n=15)<br>- Experimental group 2: Botulinum toxin B injection (n=15)	Experimental group 1 / Experimental group 2<br>**Age:** 7.73y (range 1-12) / 11.2y (range 3-17)<br>**Male:** 46.7% / 53.3%<br>**Neurological diagnosis:**<br>**Cerebral palsy:** 26.7% / 53.3%<br>**Neurodegenerative, neurometabolic or neuromuscular disorders:** 66.7% / 40.0%<br>**Undiagnosed:** 6.7% / 6.7 %
Wu et al., 2011 [28]<br>• Taiwan	**Drooling:** NR<br>**Diagnosis:** Cerebral palsy<br>**Inclusion:** A diagnosis of cerebral palsy, aged between 3 and 16 years, and a chronic drooling problem<br>**Exclusion:** Chromosomal abnormalities, progressive neurological disorder or severe concurrent illness not typically associated with cerebral palsy, active medical conditions such as epilepsy or infections, any major surgery or nerve block in the past 3 months, any known	N=20<br>- Experimental group: Botulinum toxin A injection (n=10)<br>- Control group: Placebo (normal saline injection) (n=10)	Experimental group / Control group<br>**Age:** 8.6±4.1y / 8.0±3.3y<br>**Male:** 30% / 60%<br>**Height:** 120.6±23.1cm / 116.9±19.2cm<br>**Weight:** 24.6±12.3kg / 25.2±13.3kg<br>**Type of cerebral palsy:**<br>**Diplegic**: 30% / 40%<br>**Quadriplegic**: 60% / 60%<br>**Hemiplegic**: 10% / 0%

	allergy to botulinum toxin A, and inability to chew on gauze		**GMFCS level:**<br>**Level II-III**: 60% / 50%<br>**Level IV-V:** 40% / 50%
Yilmaz et al., 2024 [29]<br>• Turkey	**Drooling:** A flow of saliva out of mouth and occurs involuntarily<br>**Diagnosis:** Cerebral palsy<br>**Inclusion:** Aged between 5 and 15 years, diagnosed with cerebral palsy with drooling complaints and having no allergic reactions to the adhesive compound of Kinesio tape<br>**Exclusion:** Using medication for drooling, having been administered botulinum toxin in the last 6 months, having active infection in the body, dysphagia, and no compliance with the treatment	N=48<br>- Experimental group: Kinesio tape (n=16)<br>- Placebo group: Sham tape (n=16)<br>- Control group: No tape (n=16)	Experimental group / Placebo group / Control group<br>**Age years:** 9.28±4.16y / 10.38±4.16y / 8.25±3.31y<br>**Male:** 56% / 44% / 63%<br>**Height:** 118.5±23.9cm / 125.6±19.4cm / 119.9±22.0cm<br>**Weight:** 23.6±11.1kg / 27.2±13.5kg / 23.5±10.8kg<br>**The number of changing bibs in pieces per day:** 2.8±3.4 / 4.0±6.7 / 2.8±2.5<br>**The number of changing clothes in pieces per day:** 1.3±1.6 / 2.2±1.4 / 1.7±2.6
Zengin et al., 2025 [30]<br>• Turkey	**Feeding problems/difficulties:** Can include poor oral hygiene tolerance, difficulty in biting and chewing, inefficient tongue movement, delays in accepting age-appropriate food consistencies, overstuffing their mouths, gagging frequently, or refusing to brush their teeth<br>**Diagnosis:** Down syndrome<br>**Inclusion:** Aged between 3-10 years with a confirmed diagnosis of Down syndrome with a feeding problem verified by a paediatrician<br>**Exclusion:** Current participation in sensory integration, occupational, or feeding/swallowing therapy, dependence on gastrostomy tube feeding and visual or hearing impairments	N=38<br>- Experimental group: Occupational therapy home programs (n=20)<br>- Control group: No intervention but provided with educational resources and guidance on accessing local therapy services (n=18)	Experimental group / Control group<br>**Age:** 6.25±1.2y / 7.23±1.5y<br>**Male:** 40% / 50%<br>**Family structure:**<br>**Nuclear family:** 80.0% / 83.3%<br>**Extended family:** 20.0% / 16.7%<br>**Primary caregiver:**<br>**Mother:** 75.0% / 77.8%<br>**Father:** 10.0% / 7.1%<br>**Other:** 15.0% / 15.1%<br>**Level of income:**<br>**Low:** 20.0% / 16.7%<br>**Lower middle:** 45.0% / 44.4%<br>**Upper middle:** 25.0% / 22.2%<br>**High:** 10.0% / 16.7%

[a]Terminology as used by author(s). *Notes*. BMI = body mass index; GMFCS = Gross Motor Function Classification System; kg = kilogram; Mo = months; NR = Not Reported; y = year(s); SD = standard deviation.

References

1. Abd-Elmonem, A.M.; Saad-Eldien, S.S.; Abd El-Nabie, W.A. Effect of oral sensorimotor stimulation on oropharyngeal dysphagia in children with spastic cerebral palsy: a randomized controlled trial. *European Journal of Physical and Rehabilitation Medicine* **2021**, *57*, 912-922, doi:10.23736/S1973-9087.21.06802-7.
2. Acar, G.; Ejraei, N.; Turkdoğan, D.; Enver, N.; Öztürk, G.; Aktaş, G. The effects of neurodevelopmental therapy on feeding and swallowing activities in children with cerebral palsy. *Dysphagia* **2022**, *37*, 800-811, doi:10.1007/s00455-021-10329-w.
3. Arvedson, J. Feeding children with cerebral palsy and swallowing difficulties. *European journal of clinical nutrition* **2013**, *67*, S9-S12.
4. Akaltun, M.S.; Umay, E.; Altindag, O.; Karaahmet, O.Z. Effectiveness of Kinesiotape and sham kinesiotape application in children with cerebral palsy with dysphagia: a randomized controlled study. *Turkish Journal of Physical Medicine and Rehabilitation* **2023**, *69*, 434-443, doi:10.5606/tftrd.2023.11066.
5. Alrefai, A.H.; Aburahma, S.K.; Khader, Y.S. Treatment of sialorrhea in children with cerebral palsy: a double-blind placebo controlled trial. *Clinical Neurology and Neurosurgery* **2009**, *111*, 79-82, doi:10.1016/j.clineuro.2008.09.001.
6. Awan, W.A.; Aftab, A.; Janua, U.I.; Ramzan, R.; Khan, N. Effectiveness of Kineso Taping with oromotor exercises in improving drooling among children with crebral palsy. *The Rehabilitation Journal* **2017**, *1*, 21-27, doi:10.52567/trj.v1i02.43.
7. Basciani, M.; Di Rienzo, F.; Fontana, A.; Copetti, M.; Pellegrini, F.; Intiso, D. Botulinum toxin type B for sialorrhoea in children with cerebral palsy: a randomized trial comparing three doses. *Developmental Medicine & Child Neurology* **2011**, *53*, 559-564, doi:10.1111/j.1469-8749.2011.03952.x.
8. Bekkers, S.; Delsing, C.P.; Kok, S.E.; van Hulst, K.; Erasmus, C.E.; Scheffer, A.R.T.; van den Hoogen, F.J.A. Randomized controlled trial comparing botulinum vs surgery for drooling in neurodisabilities. *Neurology* **2019**, *92*, e1195-e1204, doi:10.1212/WNL.0000000000007081.
9. Bekkers, S.; Pruijn, I.M.J.; van Derburg, J.J.W.; Vanhulst, K.; Kok, S.E.; Delsing, C.P.; Scheffer, A.R.T.; Vanden Hoogen, F.J.A. Surgery versus botulinum neurotoxin A to reduce drooling and improve daily life for children with neurodevelopmental disabilities: a randomized controlled trial. *Developmental Medicine & Child Neurology* **2021**, *63*, 1351-1359, doi:10.1111/dmcn.14924.
10. Berweck, S.; Bonikowski, M.; Kim, H.; Althaus, M.; Flatau-Baqué, B.; Mueller, D.; Banach, M.D. Placebo-controlled clinical trial of incobotulinumtoxinA for sialorrhea in children. *Neurology* **2019**, *97*, e1425-e1436, doi:10.1212/WNL.0000000000012573.
11. Fan, Q.-L.; Wu, Z.-F.; Yu, X.-M.; Zeng, X.-Y.; Peng, L.-S.; Su, L.-S.; Zhang, Y.-P. Clinical effect of functional chewing training in treatment of oral motor dysfunction in children with cerebral palsy: a prospective randomized controlled clinical trial. *Chinese Journal of Contemporary Pediatrics* **2020**, *22*, doi:j.issn.1008-8830.2002134.
12. Gisel, E.G.; Applegate-Ferrante, T.; Benson, J.E.; Bosma, J.F. Effect of oral sensorimotor treatment on measures of growth, eating efficiency and aspiration in the dysphagic child with cerebral palsy. *Developmental Medicine & Child Neurology* **1995**, *37*, 528-543, doi:10.1111/j.1469-8749.1995.tb12040.x.
13. Gisel, E.G. Effect of oral sensorimotor treatment on measures of growth and efficiency of eating in the moderately eating-impaired child with cerebral palsy. *Dysphagia* **1996**, *11*, 48-58, doi:10.1007/BF00385800.
14. Haberfellner, H.; Schwartz, S.; Gisel, E.G. Feeding skills and growth after one year of intraoral appliance therapy in moderately dysphagic children with cerebral palsy. *Dysphagia* **2001**, *16*, 83-96, doi:10.1007/s004550010006.

15. Inal, Ö.; Serel Arslan, S.; Demir, N.; Tunca Yilmaz, Ö. Effect of functional chewing training on tongue thrust and drooling in children with cerebral palsy: a randomised controlled trial. *Journal of Oral Rehabilitation* **2017**, *44*, 843-849, doi:10.1111/joor.12544.
16. Korbmacher, H.M.; Schwan, M.; Berndsen, S.; Bull, J.; Kahl-Nieke, B. Evaluation of a new concept of myofunctional therapy in children. *International Journal of Orofacial Myology and Myofunctional Therapy* **2004**, *30*, 40-52, doi:10.52010/ijom.2004.30.1.4.
17. Mokhlesin, M.; Mirmohammadkhani, M.; Abolfazl Tohidast, S. The effect of Kinesio Taping on drooling in children with intellectual disability: a double-blind randomized controlled study. *International Journal of Pediatric Otorhinolaryngology* **2022**, *153*, 111017, doi:10.1016/j.ijporl.2021.111017.
18. Mokhlesin, M.; Yadegari, F.; Noroozi, M.; Ravarian, A.; Ghoreishi, Z.S. Effect of action observation training on the oral phase of swallowing in children with cerebral palsy: a pilot randomized controlled trial. *Logopedics Phoniatrics Vocology* **2024**, *49*, 188-196.
19. Nordgarden, H.; Østerhus, I.; Møystad, A.; Åsten, P.; Johnsen, U.L.-H.; Storhaug, K.; Loven, J.Ø. Drooling: are botulinum toxin injections into the major salivary glands a good treatment option? *Journal of Child Neurology* **2012**, *27*, 458-464, doi:10.1177/0883073811419365.
20. Pervez, R.; Naz, S.; Babur, N.; Mumtaz, N. Effect of kinesio taping compared with manipulation therapy on drooling and speech intelligibility in children wtih oral dysphagia: a pilot study. *Alternative Therapies in Health and Medicine* **2022**, *28*, 48-51.
21. Reid, S.M.; Johnstone, B.R.; Westbury, C.; Rawicki, B.; Reddihough, D.S. Randomized trial of botulinum toxin injections into the salivary glands to reduce drooling in children with neurological disorders. *Developmental Medicine & Child Neurology* **2008**, *50*, 123-128, doi:10.1111/j.1469-8749.2007.02015.x.
22. Serel Arslan, S.; Demir, N.; Karaduman, A.A. Effect of a new treatment protocol called Functional Chewing Training on chewing function in children with cerebral palsy: a double-blind randomised controlled trial. *Journal of Oral Rehabilitation* **2017**, *44*, 43-50, doi:10.1111/joor.12459.
23. Foster, K.D.; Woda, A.; Peyron, M.-A. Effect of texture of plastic and elastic model foods on the parameters of mastication. *Journal of neurophysiology* **2006**, *95*, 3469-3479.
24. Siğan, S.N.; Uzunhan, T.; Aydinli, N.; Eraslan, E.; Ekici, B.; Çalişkan, M. Effects of oral motor therapy in children with cerebral palsy. *Annals of Indian Academy of Neurology* **2013**, *16*, 342-446, doi:10.4103/0972-2327.116923.
25. Song, W.J.; Park, J.H.; Lee, J.H.; Kim, M.Y. Effects of neuromuscular electrical stimulation on swallowing functions in children with cerebral palsy: a pilot randomised controlled trial. *Hong Kong Journal of Occupational Therapy* **2015**, *25*, 1-6, doi:10.1016/j.hkjot.2015.05.001.
26. Umay, E.; Gurcay, E.; Ozturk, E.A.; Akyuz, E.U. Is sensory-level electrical stimulation effective in cerebral palsy children with dysphagia? a randomized controlled clinical trial. *Acta Neurologica Belgica* **2020**, *120*, 1097-1105, doi:10.1007/s13760-018-01071-6.
27. Wilken, B.; Aslami, B.; Backes, H. Successful treatment of drooling in children with neurological disorders with botulinum toxin A or B. *Neuropediatrics* **2008**, *39*, 200-204, doi:10.1055/s-0028-1112115.
28. Wu, K.P.-H.; Ke, J.-Y.; Chen, C.-Y.; Chen, C.-L.; Chou, M.-Y.; Pei, Y.-C. Botulinum toxin type A on oral health in treating sialorrhea in children with cerebral palsy: a randomized, double-blind, placebo-controlled study. *Journal of Child Neurology* **2011**, *26*, 838-843, doi:10.1177/0883073810395391.
29. Yilmaz, N.; Turker, D.; Aytar, A.; Umit Yemisci, O.; Aytar, A. The acute effects of kinesio taping on drooling in children with cerebral palsy: a randomized placebo-controlled trial. *Developmental Neurorehabilitation* **2024**, *27*, 161-168, doi:10.1080/17518423.2024.2374080.

30. Zengin Yazici, G.; Akyurek, G. The effect of occupational therapy home programs on sensory processing and feeding problems in children with Down syndrome: a randomized controlled trial. *International Journal of Developmental Disabilities* **2025**, *Advance online publication*, doi:10.1080/20473869.2025.2493242.
