# Supplementary material for: Behavioural Interventions and Botulinum Toxin Injections for Drooling, Swallowing, Feeding, and Oral-Motor Outcomes in Children: A Domain-Specific Systematic Review and Meta-Analysis of Randomised Controlled Trials
Source: J Clin Med. 2026 Jun 16;15(12):4653. doi: 10.3390/jcm15124653 (PMC13302669; doi:10.3390/jcm15124653)
Supplement: Supplementary file 1 [file jcm-15-04653-s001.zip › Supplementary Table S4.pdf]

Supplementary Table S4. Outcome of behavioural interventions and botulinum toxin injections for paediatric drooling, feeding and swallowing problems.

Study	Intervention goal	Intervention agent, dosage and moments of measurements	Materials and procedures[a]	Outcome measures[b]	Treatment outcome[a]
Abd-Elmonem et al., 2021 [1]	To explore the effect of oral sensorimotor stimulation on oral-motor skills and weight gain in children with spastic quadriplegia	**Intervention agent:** Certified physical therapist/ physiotherapists<br><br>**Dosage:** 20 minutes of oral sensorimotor stimulation followed by 10 minutes rest before 90 minutes of neurodevelopmental training-based sequenced trunk co-activation exercises, five days per week for four successive months<br><br>**Moments of measurements:** NR (inferred: baseline and post-intervention at week 4)	**Oral sensorimotor stimulation:** Aims to decrease hypersensitivity of oral structures, increase jaw movement, reinforce muscle strength, improve tongue movement and enhance oral-motor organisation. Precedes mealtime and follows a structured protocol of perioral and intraoral manoeuvres based on Fucile's method. The child was placed in a semi-reclined position with neutral head and trunk alignment. Stimulation targeted cheeks, lips, gums, tongue, palate and jaw using manual massage, pressure and movement to enhance lip closure, sucking, tongue lateralisation, chewing and swallowing. Manoeuvres included cheek compression, intraoral massage, straw blowing and sucking, gum pressure, tongue stroking, palate massage, jaw movements, and chewing with small pieces of food.<br><br>**Neurodevelopmental training-based sequenced trunk co-activation exercises:** Aims to regain typical movement by prohibiting abnormal muscle tone, promoting postural reactions	**Primary outcomes:** Oral Motor Assessment Scale (OMAS)<br><br>**Secondary outcomes:** Segmental Assessment of Trunk Control (SATCo); Weight; Gross Motor Function Measure-88 (GMFM-88)	Statistically significant improvement in oral-motor skills in the experimental group ($p < 0.001$). Children in the experimental group demonstrated better mouth and lip closure, control of food and liquid during swallowing, mastication and sucking abilities. There were no statistically significant changes in the control group ($p > 0.05$).<br>Trunk control improved significantly post-treatment in both groups, but there were no statistically significant differences between the two groups post-treatment ($p > 0.05$).<br>A statistically significant post-treatment increase in weight was observed only in the experimental group ($p = 0.001$).<br>There was a statistically significant difference in post-treatment weight between the two groups ($p = 0.001$). Both groups showed statistically significant post-treatment improvements in gross motor function ($p = 0.001$), but there were no statistically significant differences between the two groups post-treatment ($p = 0.66$).

			and enhancing postural mechanisms. Focuses on restoring typical movement by inhibiting abnormal reflexes and postural malalignment and facilitating head and trunk control through positions such as prone on forearms, quadrupod, and supported sitting. Sequenced trunk co-activation exercises to facilitate functional activities by promoting coordinated activation of trunk muscles in the lateral and frontal planes during weight shifting and rotation. Righting and protective reactions were practised using physioballs and tilting boards, and functional stretching exercises were used to maintain muscle elasticity.		
Acar et al., 2022 [2]	To investigate whether neck and trunk stabilisation exercises added to feeding and oral-motor intervention will have an effect on the feeding and swallowing activity in children with cerebral palsy, and to investigate whether the exercises will increase the control of the trunk, reduce the	**Intervention agent:** Physiotherapist for NDT-B; NR for OMIS and NRCT<br><br>**Dosage:** 2 days/week for 6 weeks, for 45 minutes for a total of 12 sessions for NDT-B; for 6 weeks for NRCT; NR for OMIS	**Neurodevelopmental Therapy Method-Bobath (NDT-B):** Targeted neck and trunk stabilisation to improve postural alignment for feeding. The treatment included neutral trunk-pelvic-hip alignment with anterior-posterior and lateral weight shifts on a therapy ball, prone extension also on a therapy ball, trunk and hip extension and forward protective extension with forward weight shift, diagonal weight shift in a flexion-rotation pattern, simultaneous activation of flexors and extensors using lateral weight shift, latissimus dorsi elongation, pectoral elongation exercises, preparatory trunk activities and manual positioning and holding of the head-trunk and the mouth/lips.	**Primary outcomes:** Trunk Impairment Scale (TIS), including static sitting balance (SSB), dynamic sitting balance (DSB), coordination and total score; Schedule of Oral Motor Assessment (SOMA) for the following categories – bottle, trainer cup, cup, puree, semi-solid, solids, cracker;	SSB, DSB, and total TIS improved in both groups, and there were statistically significant interaction effects for SSB (p = 0.001) and total TIS ($p = 0.002$), but the group effect was non-significant. There were no improvements in coordination in either group, and the group effect was also non-significant.<br>There were improvements in some categories of the SOMA (trainer cup, bottle, puree), but the group effect was not statistically significant in any category.<br>The PedsQL scores improved in both groups ($p < 0.001$), but there were no statistically significant group effects ($p = 0.468$).

	duration of mealtime, reduce pain and discomfort after feeding, and increase the quality of life	**Moments of measurements:** At the start and at week 6	**Feeding and Oral-motor Intervention Strategies (OMIS):** Targeted improvements of oral-motor coordination and swallowing efficiency. Treatment included orofacial regulation, oral massage, hyolaryngeal mobilisation, extraoral stimulation, oromotor control, thermal tactile stimulation, functional chewing, tongue-lip, tongue range of motion, and soft palate exercises.<br><br>**Nutrition-Related Caregiver Training (NRCT):** Involved a home program to educate caregivers on safe and efficient feeding and nutrition management, including positioning and feeding technique, swallowing safety, appropriate containers and ingredients, food consistency, high-calorie and balanced diet, and food spillage.<br><br>The experimental group received NDT-B+OMIS+NRCT; and the control group received OMIS+NRCT only. Fiberoptic Endoscopic Evaluation of Swallowing (FEES) was used for 8 children at risk of aspiration to inform the organisation of treatment.	Pediatric Quality of Life Inventory (PedsQL)	
Akaltun et al., 2023 [3]	To investigate the short- and long-term effects of kinesio taping on dysphagia in	**Intervention agent:** A group of independent specialists	**Kinesio tape treatment:** Application of the Y-type kinesio tape by the muscle technique, and the tail part of the Y strip adhered with 10 to 15% stretching under the mandibular line to the	**Primary outcomes:** Mealtime observation of drooling, lip and tongue	In the experimental group, there were statistically significant post-treatment improvements in drooling, tongue movement, chewing, coughing/choking, and retching/vomiting as

	children with cerebral palsy	**Dosage:** Application performed twice a week for six weeks. After three days of application, the kinesio tape was removed for one day of resting, then again applied for three days<br><br>**Moments of measurement: Baseline (on the day of hospital admission), at 6 weeks (at the end of treatment), and at** 18 weeks (12 weeks after the end of treatment).	origins of the mylohyoid muscle 2.5 to 3.5cm in width, with the strip adhered to the hyoid bone just over the top to prevent direct taping of the hyoid bone. The arms of the band were glued up to the level of the manubrium sterni as a paper-off tension to prevent the facilitation of the sternohyoid muscle.<br><br>In the **sham group**, kinesio tape was applied without stretching to the suprahyoid region and did not include the origins of the mylohyoid and digastric muscles.<br><br>In addition, all participants received oral hygiene education and training in cold stimulation, head and trunk positioning, and individualised diet modifications according to their swallowing profiles.	movements, difficulty in biting, chewing and drinking, coughing/choking during/after feeding, retching/vomiting during/after feeding (measured as presence or absence); Reduced oxygen saturation during eating (measured with pulse oximetry on the second finger of the dominant hand); Functional Oral Intake Scale (FOIS); Meal time (the time it takes in minutes to swallow the last bolus starting from the first bite); Family satisfaction (study-specific rating scale five-point Likert scale)	well as FOIS score and mealtime at 6 and 18 weeks ($p < 0.017$). Family satisfaction level also improved ($p = 0.008$). There were no statistically significant improvements in lip movement, biting, drinking, or oxygen saturation.<br>In the control group, there were no statistically significant post-treatment improvements in any of the parameters measured.<br>There were statistically significant differences between the two groups in all parameters except for lip movement and biting at six weeks post-treatment. These improvements continued in drooling, drinking, coughing/choking, retching/vomiting during after/feeding, FOIS scores, and mealtime in the experimental group at 18 weeks.<br>Family satisfaction level was higher in the experimental group at six weeks (p = 0.003) but there were no statistically significant differences in family satisfaction level between the two groups at 18 weeks ($p = 0.152$).
Alrefai et al., 2009 [4]	To evaluate the efficacy and safety of local application of	**Intervention agent:** NR	**Botulinum toxin injection:** The drug was diluted with normal saline to a concentration of 20 U per 0.1 cc, and each side was injected at	**Primary outcomes:** Thomas Stonells drooling scale?	Statistically significant change in drooling frequency (from median score of 4 to 3, $p = 0.034$), in severity (from median score of 5 to 4, $p =$

	Botulinum toxin for the treatment of drooling in children with cerebral palsy compared to a placebo control	**Dosage:** A dose of 100 units of the botulinum toxin drug in the first visit and 140 units in the second visit four months later.<br><br>**Moments of measurements:** Baseline and one month after each injection	two sites, for a total of 50 U, using a 10 mm needle and the same volume in the syringe. A second set of injections was administered months after the first, with 70 Units. Anaesthesia or ultrasound guidance was not used for injection.<br><br>The control group was given a **placebo** (0.9% normal saline).	(Drooling Severity and Frequency Scale); Parent-reported side effects	0.026) and in the total score (from median score of 9 to 7) in the experimental group after the initial injection. No changes in the median scores in drooling frequency and severity in the control group after the initial injection.<br>Following the second injection, in the experimental group, 5 out of 7 had a lower total drooling score; three children who did not respond to the initial injection responded to the second injection. In the control group, one child had a decrease in the drooling score.<br>No reports of side effects after the initial or the second injection.
Awan et al., 2017 [5]	To determine the effectiveness of kinesio taping and its comparison with oral-motor exercises for the management of drooling in children with cerebral palsy	**Intervention agent:** NR<br><br>**Dosage:** For Kinesio taping, the tape was attached with 10% pull or tension and applied for a single stretch of 3 to 4 hours per day for 16 weeks; Oromotor exercises were practised for about 20 minutes two days per week for a total of 16 weeks	**Kinesio taping:** Tape was cut according to the size of the orbicularis oris muscle and applied around the mouth following the orbicularis oris muscle, with the ends of the tape overlapping each other slightly<br><br>**Oromotor exercises:** Included brushing on the tongue, gums, inner cheeks, and applying vibration on the chin and neck	**Primary outcomes:** Thomas Stonells drooling scale (Drooling Severity [DSS] and Drooling Frequency Scale DFS]); Drooling Impact Scale (DIS)	Statistically significant decrease in symptoms of drooling in both groups in terms of severity, frequency and impact of drooling (all $p < 0.05$)<br>Experimental Group 1 (Kinesio taping and oral-motor exercises) showed more early improvement in the drooling severity and impact than Experimental Group 2 (Kinesio taping only), but there were no statistically significant differences between the two groups in terms of drooling frequency.

		Moments of measurements: Week 0 (baseline), week 4, week 8, week 12 and week 16			
Basciani et al., 2011 [6]	To evaluate the efficacy and safety of three different doses of Botulinum toxin type B in reducing persistent sialorrhea in children with cerebral palsy	**Intervention agent:** NR<br><br>**Dosage:** 1500 mouse units (MU), 3000MU, and 5000 MU doses of Botulinum toxin B in low, medium and high dose groups<br><br>**Moments of measurements:** Baseline (one week before injection) and follow-up at 4 and 12 weeks	**Botulinum toxin B injection:** Bilateral injections were given into the parotid and submandibular glands with ultrasound guidance after local anaesthesia. Topical lidocaine cream was applied over the injection sites for 1 hour before the procedure. One quarter of the total dose was injected into each parotid and submandibular gland (375, 750, 1250 MU per gland for the low-, medium-, and high-dose **groups**, respectively) with a fixed injection volume of 0.25ml.	**Primary outcomes:** Thomas-Stonell drooling rating scale (Drooling Severity and Frequency Scale); Weight and number of bibs used per day.<br><br>**Secondary outcomes:** Diary of adverse effects completed by parents	No statistically significant changes in the primary outcome measures (Thomas-Stonell rating scale global scores, weight, and number of bibs) were found in the control and low-dose groups at 4 and 12 weeks. Statistically significant improvements were observed in all primary outcome measures for the medium- and high-dose groups at 4 and 12 weeks. There were no statistically significant differences in the primary outcome measures between the medium- and high-dose groups at 4 and 12 weeks. All children treated with either the medium or high dose had a reduction in sialorrhea lasting for 12 weeks.<br>Adverse effects were found only in the medium- and high-dose groups. In the medium dose group, two children (28.5%) developed dense saliva. In the high dose group, 3 children (42.8%) developed dense saliva, 1 child (14.2%) developed xerostomia, 2 children (28.5%) developed severe dysphagia and weakness, and 1 child (14.2%) developed aspiration pneumonia.
Bekkers et al., 2019 [7]	To compare the effect of submandibular	**Intervention agent:** NR	**Botulinum neurotoxin Type A (BoNT-A):** A single injection of Onabotulinum toxin A (25 U	**Primary outcomes:** Drooling Quotient	63% of children showed a clinically significant response to 2-DL after 32 weeks (vs. 26.9% for

	duct ligation (2-DL) and Botulinum toxin Type A (BoNT-A) on drooling in children and adolescents with neurodevelopmental disabilities	**Dosage:** 25 U of Onabotulinumtoxin A per submandibular gland, diluted in 0.9% saline for the BoNT-A treatment; 1% lidocaine with 1:100,000 epinephrine for the 2-DL treatment<br>**Moments of measurements:** Baseline, eight and 32 weeks post-operatively; One week after the intervention, parents were contacted about complaints and adverse events	per submandibular gland, diluted in 0.9% saline) was given under general anaesthesia. Injections were delivered into both submandibular glands using ultrasound guidance with a 25-gauge needle and a 1ml syringe.<br><br>**Submandibular duct ligation (2-DL):** The floor of the mouth is infiltrated with lidocaine 1% and epinephrine 1:100,000, then incised parallel to the frenulum, under general anaesthesia. Each submandibular duct was identified, dissected for 1-2cm, and closed with two vascular clips applied with a disposable stapler. The incision was closed with absorbable sutures. Children were given antibiotics (amoxicillin/clavulanic acid) for seven days and analgesics (paracetamol and diclofenac) for five days postoperatively.	(DQ) or caregiver's Visual Analog Scale (VAS)<br><br>**Secondary outcomes:** Drooling Severity (DS) and Drooling Frequency (DF) scales; Procedural time; Complaints as reported by caregivers during the first two weeks postoperatively; Adverse events reported by caregivers	BoNT-A), where clinical significance is defined as ^{3}50% reduction in the DQ or caregiver's VAS score; 88.9% for 2-DL and 53.8% for BoNT-A after 8 weeks.<br>The BoNT-A procedure was significantly shorter (6:13 minutes) than 2-DL (21:23 minutes).With video-evaluated DQ at 32 weeks, clinically significant responses were 72.0% for 2-DL and 26.9% to BoNT-A; and at 8 weeks, 92.6% for 2-DL and 57.7% for BoNT-A.<br>There was a statistically significant association between VAS and DQ at baseline (p = 0.039), at 8 weeks (p < 0.001) and at 32 weeks (p = 0.006).<br>Drooling severity, measured with VAS, was significantly lower after 2-DL than after BoNT-A (p < 0.001). For both treatments, VAS was significantly higher at 32 weeks than at 8 weeks (p < 0.001). This increase did not significantly differ between the two groups.<br>DQ at follow-up was 9.3% lower in the 2-DL group compared to the BoNT-A group (p =.022). For both groups, DQ was significantly higher at 32 weeks compared to at 8 weeks (p = 0.007). This increase did not significantly differ between the two groups.<br>BoNT-A did not yield a statistically significant reduction in DS after 8 (p = 0.207) or 32 weeks (p = 1.0). There was a statistically significant

					decrease in DS after 8 weeks ($p < 0.001$) but not at 32 weeks ($p = 0.061$).<br>Statistically significant reduction in DF was observed at 8 ($p = 0.001$ for BoNT-A; $p < 0.001$ for 2-DL) and 32 weeks ($p = 0.032$, $p = 0.001$, respectively).<br>There was a statistically significant difference in mean days of complaints between the two groups ($p < 0.001$), with the 2-DL group having higher mean days of complaints (9.6±3.9) than the BoNT-A group (3.1±3.6).<br>The number of adverse events was higher in the 2-DL group (40.7%) than in the BoNT-A group (19.2%), but the difference was not statistically significant ($p = 0.088$).
Bekkers et al., 2021 [8]	To compare the effect of bilateral submandibular duct ligation and botulinum neurotoxin A on drooling severity and its impact on daily life and care in children and adolescents with moderate-to-severe drooling	Same as Bekkers et al. (2019) [7]	Same as Bekkers et al. (2019) [7]	**Primary outcomes:** Study-specific caregiver questionnaire on drooling severity and impact on daily life, including multiple visual analogue scales, multiple choice questions, and open-ended questions	At 8 weeks, drooling reduced in all positions and daily activities in both groups. At 32 weeks, the degree of drooling remained decreased in all positions and daily activities for the 2-DL group ($p < 0.001$), except during walking; drooling severity did not decrease significantly during walking, intense movements, or eating, and increased significantly during drinking, talking, and strenuous activity in the BoNT-A group.<br>Excessive drooling decreased from 74% to 46% at 8 weeks after BoNT-A ($p < 0.001$) but increased to 66% at 32 weeks ($p = 0.009$). For 2-DL, excessive drooling decreased from 71% to 15% at 8 weeks ($p < 0.001$) and increased to 34% at 32

					weeks ($p < 0.001$). At both 8 and 32 weeks, 2-DL had a greater effect on excessive drooling reduction ($p < 0.001$).<br>Daily wiping decreased in both groups at 8 and 32 weeks, but the effect of 2-DL was greater than that of BoNT-A ($p = 0.002$). Hourly encouragement to swallow decreased in both groups, but the decreases at 8 and 32 weeks were statistically significant only in the 2-DL group ($p = 0.009$ and $p = 0.027$, respectively). The differences between the two groups were not statistically significant ($p = 0.655$).<br>2-DL reduced the number of bib/shawl changes at 8 weeks ($p = 0.05$) and at 32 weeks ($p = 0.039$), but BoNT-A did not reduce the number of bib/shawl changes. The differences between the two groups were not statistically significant ($p = 0.516$).<br>A statistically significant difference was found in the rate of damage to electronic devices between the two groups at 32 weeks only ($p = 0.032$), but no statistically significant group differences were found in damage to floors and/or furniture.<br>Both treatments showed a positive, though non-significant, improvement in social interactions at 8 and 32 weeks. 2-DL increased the caregiver-reported child's satisfaction with the relationship with family at 8 ($p < 0.001$) and 32 weeks (p

					= 0.015); and the child's satisfaction with life in general at 8 (*p* < 0.001) and 32 weeks (*p* = 0.003). BoNT-A yielded no statistically significant effects at 8 or 32 weeks. No statistically significant differences were found between the two groups.<br>At 32 weeks, no children in either group expressed negative reactions about appearance or negative feelings about acceptance by adults. At 32 weeks, negative reactions by peers decreased from 18% to 0% after 2-DL but increased from 16% to 30% after BoNT-A. No statistical analyses were conducted due to the limited numbers.<br>More adverse events were found in the 2-DL group than in the BoNT-A group. The total number of days with postoperative issues was greater in the 2-DL group than in the BoNT-A group.
Berweck et al., 2021 [9]	To investigate efficacy and safety of incobotulinumtoxin A (incoBoNT/A) compared with placebo for the treatment of chronic sialorrhea associated with neurologic disorders or intellectual disability in	**Intervention agent:** NR<br><br>**Dosage:** Body weight-dependent total doses of 20-75U. For patients weighing <30kg, doses were determined using a 5-weight-class dosing scheme, resulting in	**incobotulinumtoxin A (incoBoNT/A) injection:** Ultrasound-guided injections were given, with analgesics and sedatives. incoBoNT/A was dissolved in a fixed volume (at 25U/ml). The total dose was distributed in a 3:2 ratio among all parotid and submandibular glands (4 injections per session), and dose adjustments were made by adapting the volumes (parotid injections: 0.24ml to 0.9ml each; submandibular injections: 0.16ml to 0.6ml each).	**Primary outcomes:** Unstimulated salivary flow rate (uSFR), Carers' Global Impression of Change Scale (GICS); Occurrence of treatment-emergent adverse events	incoBoNT/A treatment decreased uSFR to all time points of the main period (*p* = 0.0012 at 4 weeks, *p* < 0.0001 at 8 weeks, *p* < 0.0001 at 12 weeks and *p* = 0.0003 at 16 weeks). The differences in uSFR between the two groups were statistically significant at all time points of the main period.<br>The GICS ratings were higher in the incoBoNT/A group than in the control group at all time points during the main period. The

	children or adolescents	mean doses between 1.3 and 2.2 U/kg. Patients weighing ≥30kg received a fixed dose of 75 U. The total dose was distributed in a 3:2 ratio among all parotid and submandibular glands.<br><br>**Moments of measurements:** Baseline, 4, 8 and 12 weeks after baseline and 16 weeks	The control group received equivalent volumes of **placebo** solution (0.9% sodium chloride, sucrose, human serum albumin).	overall and per injection cycle<br><br>**Secondary outcomes:** Carers' and investigators modified Teacher's Drooling Scales (mTDS); Drooling Quotient (DQ); Occurrence of treatment adverse events of special interest, serious adverse events, adverse events related to treatment, adverse events leading to discontinuation	differences in the GICS ratings between the two groups were statistically significant at all time points of the main period ($p=.032$).<br>mTDS ratings and DQs consistently showed better outcomes with incoBoNT/A compared to placebo during the main period.<br>The rates of adverse events were comparable between the two groups during the main period.<br>The secondary measures of adverse events showed low occurrences with no major differences between the two groups during the main period.<br>The findings from the extension period showed incoBoNT/A had a prolonged and sustained effect.

Fan et al., 2020 [10]	To investigate the effect of functional chewing training on improving oral-motor function in children with cerebral palsy	**Intervention agent:** Physical therapist and parents<br><br>**Dosage:** Regular continuous training for 12 weeks with 5 sessions per day, each lasting 20 minutes<br><br>**Moments of measurements:** NR (inferred: baseline and post-intervention at week 12)	**Oral-motor training (OMT):** Included passive exercises in which the lips and the tongue were moved around with the help of parents, and active exercises in which lip range of motion and tongue strengthening were trained by a physical therapist.<br><br>**Functional chewing training (FuCT):** Included providing an optimal sitting posture, placing food on the molars during each meal, stimulating lateral and rotational tongue movements, massaging the gums from incisors to the molars, and training chewing function, and gradually increasing the consistency of food.	**Primary outcomes:** Karaduman Chewing Performance Scale (KCPS); Tongue Thrust Rating Scale (TTRS); Thomas Stonells drooling scale? (Drooling Severity and Frequency Scale [DSFS])	Chewing function, tongue thrust severity, and drooling severity improved after 12 weeks of FuCT (p < 0.05), but there were no improvements in drooling frequency following FuCT ($p > 0.05$).<br>No statistically significant improvement in any of the outcome measures following OMT.<br>The degree of improvement in tongue thrust severity, drooling severity and drooling frequency was significantly greater in FuCT compared to OMT ($p < 0.05$), but there were no statistically significant differences between the two groups in chewing function ($p > 0.05$)
Gisel et al., 1995 [11]	To establish the aspiration status of children with cerebral palsy who had moderate eating impairments and to determine their response to oral sensorimotor therapy in terms of measures of growth and eating efficiency	**Intervention agent:** Feeding assistants/therapists (feeders)<br><br>**Dosage:** The treatment lasted 5-7 minutes daily before lunch, five times a week (Monday to Friday) for 20 weeks for the Experimental groups (sensorimotor)	**Sensorimotor treatment:** Targeted three main oral-motor skills, tongue lateralisation, lip control and vigour of chewing to enhance eating efficiency. Each movement was elicited using small food stimuli. Targeting tongue lateralisation involved using peanut butter on various oral surfaces to encourage lateral and protrusive tongue movements. Lip control was trained by encouraging children to close their lips around a licorice stick, blow and suck through a straw. Vigorous chewing was encouraged by placing a tea biscuit over the molars on alternating sides, with the texture resistance gradually increased.	**Primary outcomes:** Videofluoroscopy; Duration of chewing (in seconds, from the time that the food was placed in the mouth to when the first swallow was completed, using three standard food textures [puree, viscous, solid]; Duration of mealtime	26% of children aspirated, and aspiration occurred only with liquids. There were no noticeable improvements at 20 weeks after the treatment. Only one child coughed on aspiration. Two out of 7 children did not return for the post-treatment videofluoroscopy.<br>No changes in the eating duration were observed in the Experimental group and the Control group 1 (via visual inspection only). Statistically significant post-treatment decrease in the duration of eating was found in puree (p = 0.006), but an increase in the duration for viscous (p = 0.044) in the Control group 2. The

		and 10 weeks for the Control groups<br><br>**Moments of measurements:** Baseline, 10 weeks, 20 weeks	The control groups had a **school feeding routine** for 10 weeks, followed by 10 weeks of the sensorimotor treatment.	during their regular lunch time at school using the modified version of the Functional Feeding Assessment FFAm); Weight and skinfold changes	increase in the duration of chewing viscous food textures is attributed to more children in this group having advanced to more complex food textures ($p = 0.046$).<br>No statistically significant changes were found in the duration of mealtime at school. The proportion of children eating solids did not increase at a statistically significant level ($p = 0.059$).<br>No statistically significant changes were found in body weight, but there was one significant difference in the subscapular skinfold measurements during the control period (i.e. routine school feeding) for the Control group 1 ($p < 0.04$).
Gisel, 1996 [12]	To examine the efficacy of oral sensorimotor therapy in a group of moderately eating-impaired children with cerebral palsy who were all marginal oral feeders	**Intervention agent:** Instructed feeding assistants<br><br>**Dosage:** The treatment lasted 5-7 minutes daily before lunch, five times a week (Monday to Friday) for 20 weeks for the Experimental groups (sensorimotor) and the Control group 1 (chewing only); and 10	**Sensorimotor treatment:** Same as Gisel et al. (1995) [11]<br><br>**Chewing-only treatment: Children were given small pieces of fruit gelatine with** a medium-to-hard viscosity to chew for 5-7 minutes. The number of pieces to chew depended on the child's chewing ability, and they were given harder textures as they progressed.<br><br>The children in Control group 2 followed the school feeding routine for the first 10 weeks, followed by 10 weeks of sensorimotor treatment.	**Primary outcomes:** Eating time (in seconds, time required to eat three standard food textures [puree, viscous, solid]); Clearing time (in seconds, the time from first to last swallow); Duration of mealtime during their regular lunch time at school using the modified version	Within-group, no statistically significant differences were found in eating time for any food texture at 10 and 20 weeks, although there was a trend towards decreased eating time in the Experimental group (sensorimotor) and Control group 1 (chewing only). Between groups, there were no statistically significant differences.<br>There were no statistically significant between-group or within-group differences in clearing time for any food texture at 10 or 20 weeks.<br>No statistically significant differences between or within groups were found in mealtime duration during their regular lunchtime at school at 10 weeks ($p = 0.226$) or 20 weeks ($p = 0.954$).

		weeks for the Control group 2<br><br>**Moments of measurements:** Baseline, 10 weeks, 20 weeks		of the Functional Feeding Assessment (FFAm); Body weight	Children in the Experimental group showed a 2.63 percentile weight gain at week 10 but an 8.2 percentile loss at week 20; children in the Control group 1 showed a weight gain of 0.4 at weeks 10 and 0.1 gain at week 20; children in the Control group 2 showed a gain of 0.9 at week 10 and another 0.9 gain at week 20.
Haberfellner et al., 2001 [13]	To measure changes in feeding skills and growth after one year of Innsbruck Sensorimotor Activator and Regulator (ISMAR) therapy in moderately eating impaired children with cerebral palsy	**Intervention agent:** NR<br><br>**Dosage:**<br>Treatment Phase I continue wearing time of 20 minutes per day<br>Phase II; nighttime wear<br><br>**Moments of measurements:** 6 months before treatment, baseline, 6 and 12 months	**Innsbruck Sensorimotor Activator and Regulator (ISMAR) therapy:** Involved wearing the ISMAR oral appliance. ISMAR is a combination of the Andresen-Haeupl monoblock activator and the Fraenkel regulator to achieve stability of the jaw and hyoid. The occlusal shelves are held between the teeth, the gingivae, and the tongue. The lateral shelves can be extended to the molars, and the frontomedial tongue shield is placed on the tongue in a more posterior position, directing the tongue tip to an anterior point against the hard palate. During the later stages of therapy, grooves or ridges can be added to the lingual side, and a range of tongue movements can be elicited by adding small beads. The vestibular pads can be connected to the anterior medial shelves in the alveolar space.<br><br>The control group received **standard rehabilitation** at school.	**Primary outcomes:** Anthropometric measurements; modified Functional Feeding Assessment (FFAm) adapted from the Multidisciplinary Feeding Profile	Significant change in anthropometric measurements of weight and height in both groups.<br>Significant changes in spoon-feeding ($p = 0.009$), biting ($p = 0.03$), chewing ($p = 0.01$), and cup drinking ($p = 0.047$) after 12 months of intervention (phase I and II combined).<br>No significant differences in the FFAm between day and night wear.

Inal et al., 2017 [14]	To examine the effect of Functional Chewing Training on tongue thrust and drooling in children with cerebral palsy	**Intervention agent:** A physical therapist, with the assistance of parents<br><br>**Dosage:** Five sets each day for 12 weeks (each set lasting 20 minutes)<br><br>**Moments of measurements:**<br>Baseline and after 12 weeks of treatment	**Functional Chewing Training:** Involves providing optimal sitting posture for children to support oral sensorimotor functions, positioning the food to the molar area during every meal to stimulate lateral and rotational tongue movements, massage of the upper and lower gums through the front teeth to molar area, chewing training with a chewing tube to stimulate lateral and rotational tongue movements and gradually increasing the food consistency.<br><br>The control group received **classical oral-motor exercise,** including passive and active lip and tongue exercises.	**Primary outcomes:** Karaduman Chewing Performance Scale (KCPS); Tongue Thrust Rating Scale (TTRS); Thomas Stonells Drooling Scale (Drooling Severity and Frequency Scale)	At 12 weeks post-intervention, statistically significant improvements were observed in the experimental group in the chewing performance ($p = 0.001$), in tongue thrust ($p = 0.046$) and in drooling severity ($p = 0.002$), but not in drooling frequency ($p = 0.082$). No statistically significant changes in the control group in any of the measures.<br>A statistically significant difference was found between the two groups in favour of the experimental group in tongue thrust severity ($p = 0.043$).
Korbmacher et al., 2004 [15]	To evaluate any changes in the orofacial system during MFT compared with Face Former Therapy	**Intervention agent:** speech/language pathologists, dental assistants, dentists<br><br>**Dosage:** Intervention group: A training cycle consists of 20 repetitions and is performed 3 times a day. After a three-week training period, the appliance is worn overnight<br>Control group: NR	**Face Former therapy**: consists of tongue and lip exercises using a flexible silicone appliance (Face Former, Akkuphon®, Unna, Germany. The device is inserted into the oral vestibulum, behind the lips and in front of the teeth. The basic exercise consists of actively compressing the lip wedge with the lips for 6 seconds, followed by a 6-second relaxation phase.<br><br>**Conventional myofunctional therapy:** stipulated individually by the therapist and based on the approaches of Kittel, Grums and Garliner.	**Primary outcomes:** Clinical examination using a standardised diagnostic sheet for patients with orofacial dysfunctions (Korbmacher and Kahl Nieke, 2001): (mouth) breathing (clinical observation), lip strength (Myo-Bar-Meter: Akkuphon, Unna, Germany), palaeography (swallowing	Significant decrease of mouth breathing in the study group at six months after the start ($p = 0.001$), but not in the control group ($p = .097$)<br>A statistically significant increase in lip strength over the three time points was established in both groups, but no statistically significant difference between groups.<br>Regarding the swallowing pattern, the intervention group showed a statistically significant improvement ($p = .000$), whereas the control group showed no statistically significant improvement ($p = .097$).<br>No statistically significant improvement was found in sigmatism

		**Moments of measurements:** Baseline, three and six months after the start		pattern), sigmatism (visual/acoustic rating); Extraoral and intraoral clinical photos	
Mokhlesin et al., 2022 [16]	To investigate the effect of adding Kinesio Taping (KT) to traditional oral-motor treatment (OMT) for drooling management in children with intellectual disability	**Intervention agent:** speech-language pathologist and caregiver<br><br>**Dosage:** KT involved 10%-15% tension for orbicularis oris muscle and 25% tension for masseter, geniohyoid muscles, and taped for 1 hour in the first week, and increased weekly by a half for 4 weeks in total; OMT was performed 5 days per week, twice a day (once by a speech-language pathologist, and once by a caregiver) for 5	**Oral-motor treatment (OMT):** Applied as a traditional method to include vibration of masseter muscles, suprahyoid muscles in the submandibular triangle, and orbicularis oris muscles. Brushing, icing of the tongue, stroking and tapping of the lips and resistance training of the lips and the tongue were also given.<br>**Kinesio Taping (KT):** Water-resistant EPOS TAPE model with 5cm width was applied on orbicularis oris, masseter, and geniohyoid muscles from the muscles' origins to insertion. Two I-tapes were applied at the centre of the mouth (one above the upper lip and one below the lower lip). A 2.5cm-wide tape was applied to the masseter muscles. The distance from the zygomatic arch to the mandibular angle was measured with the mouth open, then two I-shaped tabs were cut and applied to the zygomatic arch at the top. An I-tape with a 2.5cm width was applied to the mandible to the hyoid cartilage while the head was in extension posture.	**Primary outcomes:** Thomas Stonells drooling scale (Drooling Severity and Frequency Scale [DRS]); Drooling Quotient (DQ): salivary outflow rate	Both groups improved in DQ after the treatment, but the improvement was greater in the experimental group ($p = 0.008$), with a moderate effect size (0.62).<br>DRS scores reduced in both groups after the treatment, and there was no statistically significant post-treatment difference between the two groups ($p > 0.05$).

		minutes also for 4 weeks<br><br>**Moments of measurements:** Post-treatment measure one day after the last therapy session	The experimental group received both **OMT and KT**; the control group received **OMT and the sham** treatment, in which taping was applied without any tension.		
Mokhlesin et al. 2024 [17]	To investigate the impact of Action Observation Training (AOT) on the oral phase of swallowing in children with spastic CP	**Intervention agent:** Speech language pathologist<br><br>**Dosage**: 10 weeks; 5 days a week; once a day for 20 minutes.<br>Follow-up measurement 4 weeks after finishing the intervention<br><br>**Moments of measurement:** pre-intervention, post-intervention, and at one month of follow-up.	**Action observation-oral sensorimotor therapy (AOOST):** positioning, action observation, and oral sensorimotor therapy (an oral-motor swallowing task was practised for 2 min using foods and feeding utensils for sensory stimulation). The movement was demonstrated, and tactile/kinetic prompts were used to reinforce it.<br><br>**Action observation:** watching four different videos for each of the targeted oral skills: a) maintain lip seal around the spoon, b) performing lateral tongue movements, c) chewing, and d) biting, followed by the chewing and swallowing sequence (3 min. each)<br><br>**Control group:** watching videos unrelated to eating and swallowing movements. These included body gestures, interviews, cooking, and naming. (3 min. each)	**Primary outcomes:** Schedule of Oral Motor Assessment (SOMA);<br>Oral Motor Assessment Scale (OMAS)<br><br>**Secondary outcomes** Pedi-Eating Assessment Tool (EAT Pediatric); Feeding and Swallowing Impact Survey (FS-IS)	Statistically significant difference in SOMA scores between the two groups at the post-intervention assessment (*p* = 0.03), but there was no significant difference in the follow-up evaluation after one month (*p* = 0.09).<br>Within-group analysis indicated significant differences in SOMA scores among the three time points in both the intervention and control groups (p =.001 and *p* = 0.001, respectively.<br>Significant difference in OMAS scores between the two groups in both post-intervention and follow-up evaluations, with *p* = 0.03 and *p* = 0.04, respectively.

Nordgarden et al., 2012 [18]	To compare the effects and adverse effects of botulinum toxin A injections to both the parotid and submandibular glands with similar injections to the submandibular glands only.	**Intervention agent:** ENT specialist with a radiologist<br><br>**Dosage:** 25 IU botulinum toxin A in 0.8 mL fluid was injected into each gland; the total dose was 100 IU for the injection in 4 glands<br><br>**Moments of measurements**: at baseline, 8 weeks and 12 weeks posttreatment	**Botulinum toxin A injections**: The injections were performed as a day-surgery procedure under general anaesthetic, with ultrasound guidance using a needle guide attached to a probe. The needle was placed centrally in the gland tissue. 25 IU botulinum toxin A (Botox, Allergan) in 0.8 mL fluid was injected into each gland.<br><br>Experimental group 1 received injections to both the **parotid and submandibular salivary gland**s, and Experimental group 2 received injections to the **submandibular salivary glands** only.	**Primary outcomes:** Drooling coefficient, rate of saliva secretion; Self- or parent-rated impact of drooling on quality of life; Self- or parent-rated adverse effects	Two children showed decreases in the drooling quotient, the rate of saliva secretion, and the impact of drooling on their quality of life.<br>Two children had an increased drooling quotient, but a decrease in measured saliva secretion and the influence of drooling on their quality of life<br>One child had a decrease in both the drooling quotient and measured saliva secretion, but a slight increase in the influence of drooling on the quality of life.<br>The adverse effects observed included dysphagia, dysarthria, and increased viscosity of saliva. All participants but one demonstrated an increase in the dental plaque index.
Pervez et al., 2022 [19]	To determine the effectiveness of Kinesio Taping (KT) and Manipulation Therapy (MT) on drooling and speech intelligibility in children with oral dysphagia	**Intervention agent:** NR<br><br>**Dosage:** 5 days a week for 20 sessions in total. Each session was 45 minutes.<br>Control group sessions were provided 5 days a week and were 3x 15 minutes a day	**Kinesio taping (KT):** cutting 2 “I” tapes according to the structure of the patient’s lip muscles. While maintaining 10% tension, the tape was applied to the muscles in the corner of the upper lip with the mouth fully open.<br><br>**Manipulation therapy (MT)**: oral-motor manipulation therapy including: tapping around all the lip muscles (orbicularis oris) for 5 minutes (150 times), finger massage around the lip muscles: starting from the midline of the lips, holding both lips with 2 fingers of both hands,	**Primary outcomes:** Modified Teachers’ Drooling Scale (mTDS); Intelligibility Rating Scale (1-7) (speech intelligibility)	KT and MT both significantly improved drooling severity, with large (p = 0.004; r = -0.89) and medium (p = 0.025; r = -0.70) effects, respectively.<br>Speech intelligibility significantly improved in the KT group (p = 0.014; r = -0.77), but in the MT group, there was no statistically significant change (p = 0.317).<br>No significant difference between the KT and MT groups regarding drooling severity (p = 0.06) and speech intelligibility (p = 0.07) after 1 month

		**Moments of measurements:** At baseline and at one month	then towards the jaw bone, and around the base of the tongue with the help of the thumb for 5 minutes massage, and rhythmic pressure		
Reid et al., 2008 [20]	To assess the effectiveness of botulinum toxin A injections into the submandibular and parotid glands on drooling in children with cerebral palsy and other neurological disorders<br><br>To ascertain the duration of any effect and the timing of the maximal response	**Intervention agent:** NR<br><br>**Dosage:** A total of 100 units of botulinum toxin A were diluted in 4ml of normal saline, and 1ml was injected into the centre of each salivary gland (i.e., 25 units/gland), or at a dose of 4 units/kg if the patient weighed less than 25kg.<br><br>**Moments of measurements:** Baseline and 1-month	**Botulinum toxin A injections into the submandibular and parotid glands:** Under a short general anaesthetic and ultrasonic guidance, botulinum toxin A was injected into the centre of each salivary gland.<br><br>The control group received **no treatment**.	**Primary outcomes:** Drooling Impact Scale (DIS=DrI: 10 questions on semantic differential scale); Shortened scale (3 questions related to severity and frequency of drooling and number of bibs); Perceived effects of injections	Statistically significant difference in the Drooling Impact Scale score between the groups at 1-month postinjection [Control: 61.74 (12.35) vs. Experimental: 34.29 (14.96), 95% confidence interval for difference: 19.37 to 35.52]. The most significant changes were in the frequency and severity of drooling, and the number of times bibs or clothing needed to be changed due to excessive drooling.<br>Four children had no response to the injection at 1 month, and another four children had a mediocre response with a 10-20 points reduction (DIS).<br>The difference remained significant at 6 months post-injection, and the maximal response occurred at 1 month post-injection.<br>One family reported difficulties with swallowing, choking, and deterioration of speech for the first 5 days after the injection.

		postinjection/postbaseline for the Drooling Impact Scale<br>Monthly from 2-6 months and at 1 year for the intervention group on the shortened scale			Another child developed a severe chest infection on day 5, and a third child had his first seizure 2 days after the injection. No<br>acute problem was reported in the control group.
Serel Arslan et al., 2017 [21]	To investigate the effect of functional chewing training (FuCT) on chewing function in children with cerebral palsy.	**Intervention agent:** parents trained by a physiotherapist<br><br>**Dosage:**<br>5x20 minutes daily, 5 days a week, for 12 weeks<br><br>**Moments of measurements:** Baseline and after 12 weeks	**Functional chewing training (FuCT):** A holistic approach including treatment strategies, including impairment-based (positioning the child and food, sensory stimulation and chewing exercise) and adaptive (food consistency) components.<br><br>The control group received **traditional oral-motor exercises,** including passive and active exercises of lips and tongue.	**Primary outcomes:** Behavioral Pediatrics<br>Feeding Assessment Scale (BPFAS);<br>Karaduman Chewing Performance Scale (KCPS)	A significant difference was found between the FuCT and control groups in favour of the FuCT group for KCPS levels and BPFAS subscales, except for the restriction score after 12 weeks of intervention ($p < 0.001$).<br>The FuCT group showed improvement in chewing performance according to the KCPS ($p < 0.001$) and in feeding behaviours according to the BPFAS ($p < 0.001$). The control group did not show any improvement in chewing performance (p =.07) but presented improvement in four subscales of the BPFAS, namely, total frequency score ($p = 0.02$), total problem score ($p = 0.03$), child frequency score (p =. 02), and child problem score (p =.01).
Siğan et al., 2013 [22]	To assess the effect of oral-motor therapy on oral-motor functions and neuromotor development in	**Intervention agent:** Physiotherapist<br><br>**Dosage:** 1 hour session once a week for 6	**Oral-motor therapy:** Involved increasing the tactile and proprioceptive aspects of eating. Food texture was gradually thickened to improve mouth function and control. Mouth control was performed when muscle control was insufficient. Manual oral stimulation was	**Primary outcomes:** Status of swallowing, gag and asymmetric tonic neck (ASTN) reflexes; Oral-motor	No statistically significant differences in ASTN ($p > 0.05$), sucking difficulty ($p = 0.248$), or coughing/suffocation improvement ($p > 0.05$).<br>Statistically significant improvements in chewing, swallowing, drooling, independent feeding and feeding problems, swallow delay,

	children with cerebral palsy	months for a total of 12 sessions<br><br>**Moments of measurements:** Before and after treatment	provided. For drinking, children were taught correct midline hand use and the correct class-use technique with moderately dense liquids. Mouth control, positioning and posture control were also taught to reduce drooling. Spoon feeding and proper positioning were taught to families.<br><br>The control group received **routine physiotherapy**.	assessment form (presence and absence of oral-motor functions); Functional Feeding Assessment (FFA) subscale of the Multidisciplinary Feeding Profile (MFP); Bayley Scales of Infant Development (BSID-II)	aspiration, and choking in the Experimental group ($p < 0.05$).<br>No statistically significant differences in jaw lateralisation ($p > 0.05$), but statistically significant improvements in tongue extension, elevation, and lateralisation; jaw stabilisation, spoon feeding, lip wiping, and holding mouth/lips closed, tolerated food texture and swallowing evaluation results in the Experimental group ($p < 0.05$).<br>Statistically significant improvements in drooling in the Experimental group ($p < 0.05$).<br>FFA scores improved statistically significantly more in the Experimental group than in the Control group ($p < 0.05$).<br>The BSID-II scores improved in both groups ($p < 0.05$).
Song et al., 2015 [23]	To determine the therapeutic effects of oral sensorimotor treatment (OST) and Neuromuscular electrical stimulation (NMES) on oral-motor functions in children with CP and dysphagia.	**Intervention agent:** Occupational therapist<br><br>**Dosage:** Twice a week for 8 weeks, 10 minutes of OST and 20 minutes of NMES/sham<br><br>**Moments of measurements:** pre- and post-treatment	**Oral sensorimotor treatment (OST)** included various<br>sensory stimuli to the cheeks, chin, lips, tongue, and oral palate using human fingers, a vibrator, and an ice stick 10 times each.<br><br>**Neuromuscular electrical stimulation (NMES)** with a dual-channel device (Simplus DP 200; Cybermedic Corp., Iksan, South Korea). The parameters of electrical stimuli were 80 Hz, 300 milliseconds, with a 1-second interval. For channel 1, two sets of electrodes were placed horizontally over the throat, between the jaw and	**Primary outcomes:** Behavioral Assessment Scale of Oral Functions in Feeding (BASOFF); American Speech, Language & Hearing Association's National Outcomes Measurement System swallowing scale (ASHA NOMS)	Significant increase of the BASOFF score in both groups ($p < 0.05$). The NMES group demonstrated significant<br>improvement in all subcategories, while the control group showed no significant improvement in three subcategories (jaw closure, sipping liquid, and swallowing liquid<br>without excess loss) ($p < 0.05$). The NMES group demonstrated<br>a significantly greater improvement than the control group in lip closure while swallowing, swallowing food without excess loss, sipping liquid, swallowing liquid without excess loss,

			hyoid, approximately over the belly of the digastric muscles. For channel 2, two additional sets of electrodes were placed horizontally between the hyoid and the thyroid notch, approximating the infrahyoid muscles. The current intensity was determined by palpation for muscle contraction. The typical current level ranged from 3 mA to 5 mA.<br><br>The experimental group received OST and an additional 20-minute treatment with NMES, and the control group received OST and **sham NMES**		swallowing without cough, and total score ($p < 0.05$).<br>No significant change between or within the groups in the ASHA NOMS swallowing scale.
Umay et al., 2020 [24]	To determine the effects of sensory-level electrical stimulation treatment applied to bilateral masseter muscles at the lowest current level combined with conventional dysphagia rehabilitation	**Intervention agent:**<br>Physical Medicine and Rehabilitation specialist<br><br>**Dosage:**<br><br>30 min/day, 5 days/week, for 4 weeks, two pieces of<br>3 × 3 cm surface electrodes were placed; one on the ramus of the mandible and one on the bell of the masseter muscle (the chin is in	**Electric stimulation:** Two pieces of 3 × 3 cm surface electrodes were placed; one on the ramus of the mandible and the other on the masseter muscle bell (the chin is in intercuspal position and in the midline). The current stimulation intensity was established by determining the threshold sensitivity using an incremental protocol and stabilising it during the treatment session.<br><br>**Conventional dysphagia rehabilitation**: involves diet, nutrition, strengthening exercises of oropharyngeal muscles, posture and environmental modifications, swallowing manoeuvres and specific movement-based techniques,	**Primary outcomes:**<br>Pediatric Eating Assessment Tool-10 (Pedi-Eat-10); Flexible Fiberoptic Endoscopic Evaluation of Swallowing (FEES)<br><br>**Secondary outcomes** Physical examination and feeding observation: drooling, lip and tongue movement, biting, chewing, eating small/large food	Significant changes for the intra-group treatment in both groups for Pedi-Eat-10 (Experimental group, p = 0.001; Control group, p = 0.007); the changes obtained with FEES were significant only for the experimental group (p = 0.003).<br>Significant improvement with treatment was observed in Pedi-Eat-10 and dysphagia level in the experimental group compared to the control group 2 (p = 0.001 and p = 0.042, respectively).<br>Significant differences in drooling (p =.011), tongue movements (p =.018), chewing (p = 0.017), and eating large foods (p = 0.001) in favour of the experimental group.

		intercuspal position and in the midline)<br><br>**Moments of measurements:** Baseline and at 4 weeks post-treatment	thermal, tactile, and pressure stimulation to enhance sensory input<br><br>The control group received **sham stimulation** with the electrodes	ability, drinking liquid and eating food ability, oral food accumulation, repeated swallowing/head tilt, nasal regurgitation, coughing/choking with feeding, retching/vomiting with feeding, voice changes, O_2 saturation, feeding duration (≥ 45 min), food refusal	
Wilken et al., 2008 [25]	To examine the safety, application and optimal dosage of botulinum toxin A or B injections in the salivary glands with ultrasound guidance in children with neurological disorders.	**Intervention agent:** NR<br><br>**Dosage:** Start: 100 U / kg (botulinum toxin B) or 80 MU (botulinum toxin A). The dosage was increased to 120 U/kg and 100 MU, respectively. Dosage was divided into 3 injections on both sides, two in the parotid gland (one in	**Botulinum toxin A injections**: Start with 80 MU of Botulinum toxin A (Botox®, Pharm-Allergan, Ettlingen, Germany). Botox® was reconstituted with 2 mL of 0.9 % sodium chloride solution. **Group Botulinum toxin B**; Started at 100 U/kg (NeuroBloc®, Elan Pharma USA). Neurobloc® was already in solution in 1 mL or 1.5 mL, depending on the total dosage.<br><br>In single cases, local anaesthesia was used (20 % Emla ® (1 g contains 25 mg lidocaine and 25 mg prilocaine, AstraZeneca, Wedel, Germany)	**Primary outcomes:** Teachers Drooling Scale (TDS); Study-specific parental questionnaire	29 patients responded with a reduction of TDS score of 1 or 2 in the parent's questionnaire, one child treated with botulinum toxin A did not show any reduction of sialorrhea in the TDS and four children with botulinum toxin B. Group A improved by a total of 39 points on the TDS, whereas group B improved 32 points, four weeks after the last treatment.

		front of the isthmus, the other one below) (30 – 40 U / kg botulinum toxin B – 25 – 35 MU total dose of botulinum toxin A) and one injection into the submandibular gland (20 U / kg botulinum<br>toxin B – 15 MU total dose of botulinum toxin A).<br><br>**Moments of measurements:** Four weeks after the injection			
Wu et al., 2011 [26]	To determine the effects of ultrasonography-controlled, low-dose botulinum toxin Type A injections for drooling, concentrate saliva compositions, e oral pH, and cariogenic<br>bacterial counts	**Intervention agent:** Experienced paediatric physiatrist<br><br>**Dosage:** Botulinum toxin type A reconstituted with 1 mL of saline (0.9% sodium chloride solution) to a concentration of 10 U/0.1 mL. The	**Botulinum toxin A injections:** The injection was set 1 to 1.2 cm behind the mandibular ramus for the parotid gland and at the midpoint of the submandibular gland, as seen on the longitudinal scan. Injection was controlled sonographically with a 30-gauge needle.<br><br>The control group was given a **placebo** (saline solution) injected in the same locations.	**Primary outcomes:** Subjective Drooling Scale (drooling severity, bibs change scale); Saliva flow rate; Saliva composition analysis (pH, protein, calcium, phosphate, potassium ions); Salivary	Significant decrease in salivary flow rate in the botulinum toxin type A group at the 1-month (p = 0.037) and 3-month (p = 0.041) follow-up compared to the control group.<br>No differences in the subjective drooling scales in both groups, nor at 1 month, nor at 3 months after the injection. No significant difference between the saliva compositions (salivary pH, protein, potassium, calcium, and phosphate contents, in changes in S mutans and the lactobacilli colony count was noted before and after

		total dose was adapted to the body weight. The maximum dose for each submandibular gland was 10 U, and no participant received more than 50 U in total.<br><br>**Moments of measurements:** Prior to and one and three months after the injection		Cariogenic Bacterial Analysis	injection between the 2 groups and in the comparison of the follow-up assessments
Yilmaz et al., 2024 [27]	To determine the short-term effects of Kinesio taping on drooling in children with cerebral palsy	**Intervention agent:** Physiotherapist specialised in Kinesio taping and trained parents<br><br>**Dosage:** Continued application of the tape for 2 days<br><br>**Moments of measurements**: at baseline, after 45 minutes and after two days of the application	**Kinesio tape treatment:** Application of kinesiology tape (I-tape) along the orbicularis oris muscle using the mechanical correction technique with 50-75% tension, and an I-tape with a 50-75% tension using the mechanical correction technique to the suprahyoid region. An application was made by a physiotherapist specialised in Kinesio taping. Trained parents continue to apply the tape for 2 days.<br><br>I-tape approximately 5 cm long was applied to the cheek without any tension for the **placebo** group.	**Primary outcomes:** Thomas Stonells drooling scale? (Drooling Severity and Frequency Scale); 5-minute Drooling Quotient method	Statistically significant improvements in the experimental group in drooling severity, frequency and amount 45 minutes post-intervention and 2 days post-intervention ($p < 0.01$) and no statistically significant difference in drooling severity, frequency and amount in the placebo and control groups 45 minutes post-intervention and 2 days post-intervention.

Zengin et al., 2025 [28]	To examine if occupational therapy home programs (OTHPs) are effective in improving sensory processing functions in children with Down syndrome.<br><br>To examine whether OTHPs are effective in addressing feeding problems in children with Down syndrome	**Intervention agent:** Occupational therapist<br><br>**Dosage:** An eight-week program, 30-45 minutes per day<br><br>**Moments of measurements:** at **the** start and after 8 weeks	**Occupational therapy home programs (OTPH):** A 5-step structured process with an individual-designed intervention concerning sensory-based pre-feeding activities, structured play routines, food exposure strategies, and parent-led implementation principles for goals on oral sensory tolerance, food selectivity, chewing and swallowing, food refusal and self-feeding skills. Specific activities were selected according to each child's sensory needs, feeding history, and developmental level. Parents were instructed and coached.<br><br>The control group received **alternative support** with educational resources on sensory processing and feeding, and guidance on accessing local therapy services.	**Primary outcomes:** Turkish version of the Sensory Profile (original version Dunn, 2006) [29]; Screen Tool of Feeding Problems (STEP)	Significant results in the OTHP (experimental) group on oral sensory processing and touch processing ($p < 0.001$). In the non-OTHP group, there were no significant differences between pre- and post-intervention on sensory processing scores in any subdomain ($p > 0.05$).<br>Food selectivity, feeding skills, food refusal behavioural problems, and nutrition-related behavioural problems scores increased significantly in the OTHP group ($p <.001$). The aspiration risk did not change significantly ($p > .05$). In the non-OTHP group, no significant differences were found in the STEP total or subdomain scores between pre- and post-intervention assessments ($p >.05$).

[a] Terminology as used by authors; [b] Primary and secondary outcomes as reported by authors. *Notes*. ENT = Ear Nose Throat; TDS = Teacher/Teachers/Teachers'/Teacher's Drooling Scale.

References

1. Abd-Elmonem, A.M.; Saad-Eldien, S.S.; Abd El-Nabie, W.A. Effect of oral sensorimotor stimulation on oropharyngeal dysphagia in children with spastic cerebral palsy: a randomized controlled trial. *European Journal of Physical and Rehabilitation Medicine* **2021**, *57*, 912-922, doi:10.23736/S1973-9087.21.06802-7.
2. Acar, G.; Ejraei, N.; Turkdoğan, D.; Enver, N.; Öztürk, G.; Aktaş, G. The effects of neurodevelopmental therapy on feeding and swallowing activities in children with cerebral palsy. *Dysphagia* **2022**, *37*, 800-811, doi:10.1007/s00455-021-10329-w.
3. Akaltun, M.S.; Umay, E.; Altindag, O.; Karaahmet, O.Z. Effectiveness of Kinesiotape and sham kinesiotape application in children with cerebral palsy with dysphagia: a randomized controlled study. *Turkish Journal of Physical Medicine and Rehabilitation* **2023**, *69*, 434-443, doi:10.5606/tftrd.2023.11066.
4. Alrefai, A.H.; Aburahma, S.K.; Khader, Y.S. Treatment of sialorrhea in children with cerebral palsy: a double-blind placebo controlled trial. *Clinical Neurology and Neurosurgery* **2009**, *111*, 79-82, doi:10.1016/j.clineuro.2008.09.001.
5. Awan, W.A.; Aftab, A.; Janua, U.I.; Ramzan, R.; Khan, N. Effectiveness of Kineso Taping with oromotor exercises in improving drooling among children with crebral palsy. *The Rehabilitation Journal* **2017**, *1*, 21-27, doi:10.52567/trj.v1i02.43.
6. Basciani, M.; Di Rienzo, F.; Fontana, A.; Copetti, M.; Pellegrini, F.; Intiso, D. Botulinum toxin type B for sialorrhoea in children with cerebral palsy: a randomized trial comparing three doses. *Developmental Medicine & Child Neurology* **2011**, *53*, 559-564, doi:10.1111/j.1469-8749.2011.03952.x.
7. Bekkers, S.; Delsing, C.P.; Kok, S.E.; van Hulst, K.; Erasmus, C.E.; Scheffer, A.R.T.; van den Hoogen, F.J.A. Randomized controlled trial comparing botulinum vs surgery for drooling in neurodisabilities. *Neurology* **2019**, *92*, e1195-e1204, doi:10.1212/WNL.0000000000007081.
8. Bekkers, S.; Pruijn, I.M.J.; van Derburg, J.J.W.; Vanhulst, K.; Kok, S.E.; Delsing, C.P.; Scheffer, A.R.T.; Vanden Hoogen, F.J.A. Surgery versus botulinum neurotoxin A to reduce drooling and improve daily life for children with neurodevelopmental disabilities: a randomized controlled trial. *Developmental Medicine & Child Neurology* **2021**, *63*, 1351-1359, doi:10.1111/dmcn.14924.
9. Berweck, S.; Bonikowski, M.; Kim, H.; Althaus, M.; Flatau-Baqué, B.; Mueller, D.; Banach, M.D. Placebo-controlled clinical trial of incobotulinumtoxinA for sialorrhea in children. *Neurology* **2019**, *97*, e1425-e1436, doi:10.1212/WNL.0000000000012573.
10. Fan, Q.-L.; Wu, Z.-F.; Yu, X.-M.; Zeng, X.-Y.; Peng, L.-S.; Su, L.-S.; Zhang, Y.-P. Clinical effect of functional chewing training in treatment of oral motor dysfunction in children with cerebral palsy: a prospective randomized controlled clinical trial. *Chinese Journal of Contemporary Pediatrics* **2020**, *22*, doi:j.issn.1008-8830.2002134.
11. Gisel, E.G.; Applegate-Ferrante, T.; Benson, J.E.; Bosma, J.F. Effect of oral sensorimotor treatment on measures of growth, eating efficiency and aspiration in the dysphagic child with cerebral palsy. *Developmental Medicine & Child Neurology* **1995**, *37*, 528-543, doi:10.1111/j.1469-8749.1995.tb12040.x.
12. Gisel, E.G. Effect of oral sensorimotor treatment on measures of growth and efficiency of eating in the moderately eating-impaired child with cerebral palsy. *Dysphagia* **1996**, *11*, 48-58, doi:10.1007/BF00385800.
13. Haberfellner, H.; Schwartz, S.; Gisel, E.G. Feeding skills and growth after one year of intraoral appliance therapy in moderately dysphagic children with cerebral palsy. *Dysphagia* **2001**, *16*, 83-96, doi:10.1007/s004550010006.

14. Inal, Ö.; Serel Arslan, S.; Demir, N.; Tunca Yilmaz, Ö. Effect of functional chewing training on tongue thrust and drooling in children with cerebral palsy: a randomised controlled trial. *Journal of Oral Rehabilitation* **2017**, *44*, 843-849, doi:10.1111/joor.12544.
15. Korbmacher, H.M.; Schwan, M.; Berndsen, S.; Bull, J.; Kahl-Nieke, B. Evaluation of a new concept of myofunctional therapy in children. *International Journal of Orofacial Myology and Myofunctional Therapy* **2004**, *30*, 40-52, doi:10.52010/ijom.2004.30.1.4.
16. Mokhlesin, M.; Mirmohammadkhani, M.; Abolfazl Tohidast, S. The effect of Kinesio Taping on drooling in children with intellectual disability: a double-blind randomized controlled study. *International Journal of Pediatric Otorhinolaryngology* **2022**, *153*, 111017, doi:10.1016/j.ijporl.2021.111017.
17. Mokhlesin, M.; Yadegari, F.; Noroozi, M.; Ravarian, A.; Ghoreishi, Z.S. Effect of action observation training on the oral phase of swallowing in children with cerebral palsy: a pilot randomized controlled trial. *Logopedics Phoniatrics Vocology* **2024**, *49*, 188-196.
18. Nordgarden, H.; Østerhus, I.; Møystad, A.; Åsten, P.; Johnsen, U.L.-H.; Storhaug, K.; Loven, J.Ø. Drooling: are botulinum toxin injections into the major salivary glands a good treatment option? *Journal of Child Neurology* **2012**, *27*, 458-464, doi:10.1177/0883073811419365.
19. Pervez, R.; Naz, S.; Babur, N.; Mumtaz, N. Effect of kinesio taping compared with manipulation therapy on drooling and speech intelligibility in children wtih oral dysphagia: a pilot study. *Alternative Therapies in Health and Medicine* **2022**, *28*, 48-51.
20. Reid, S.M.; Johnstone, B.R.; Westbury, C.; Rawicki, B.; Reddihough, D.S. Randomized trial of botulinum toxin injections into the salivary glands to reduce drooling in children with neurological disorders. *Developmental Medicine & Child Neurology* **2008**, *50*, 123-128, doi:10.1111/j.1469-8749.2007.02015.x.
21. Serel Arslan, S.; Demir, N.; Karaduman, A.A. Effect of a new treatment protocol called Functional Chewing Training on chewing function in children with cerebral palsy: a double-blind randomised controlled trial. *Journal of Oral Rehabilitation* **2017**, *44*, 43-50, doi:10.1111/joor.12459.
22. Siğan, S.N.; Uzunhan, T.; Aydinli, N.; Eraslan, E.; Ekici, B.; Çalişkan, M. Effects of oral motor therapy in children with cerebral palsy. *Annals of Indian Academy of Neurology* **2013**, *16*, 342-446, doi:10.4103/0972-2327.116923.
23. Song, W.J.; Park, J.H.; Lee, J.H.; Kim, M.Y. Effects of neuromuscular electrical stimulation on swallowing functions in children with cerebral palsy: a pilot randomised controlled trial. *Hong Kong Journal of Occupational Therapy* **2015**, *25*, 1-6, doi:10.1016/j.hkjot.2015.05.001.
24. Umay, E.; Gurcay, E.; Ozturk, E.A.; Akyuz, E.U. Is sensory-level electrical stimulation effective in cerebral palsy children with dysphagia? a randomized controlled clinical trial. *Acta Neurologica Belgica* **2020**, *120*, 1097-1105, doi:10.1007/s13760-018-01071-6.
25. Wilken, B.; Aslami, B.; Backes, H. Successful treatment of drooling in children with neurological disorders with botulinum toxin A or B. *Neuropediatrics* **2008**, *39*, 200-204, doi:10.1055/s-0028-1112115.
26. Wu, K.P.-H.; Ke, J.-Y.; Chen, C.-Y.; Chen, C.-L.; Chou, M.-Y.; Pei, Y.-C. Botulinum toxin type A on oral health in treating sialorrhea in children with cerebral palsy: a randomized, double-blind, placebo-controlled study. *Journal of Child Neurology* **2011**, *26*, 838-843, doi:10.1177/0883073810395391.
27. Yilmaz, N.; Turker, D.; Aytar, A.; Umit Yemisci, O.; Aytar, A. The acute effects of kinesio taping on drooling in children with cerebral palsy: a randomized placebo-controlled trial. *Developmental Neurorehabilitation* **2024**, *27*, 161-168, doi:10.1080/17518423.2024.2374080.
28. Zengin Yazici, G.; Akyurek, G. The effect of occupational therapy home programs on sensory processing and feeding problems in children with Down syndrome: a randomized controlled trial. *International Journal of Developmental Disabilities* **2025**, *Advance online publication*, doi:10.1080/20473869.2025.2493242.

29. Dunn, W. Sensory Profile Supplement: User’s Manual Blooming-ton. *MN: Pearson* **2006**.
